# Targeted inhibition of the HNF1A/SHH axis by triptolide overcomes paclitaxel resistance in non-small cell lung cancer

**DOI:** 10.1038/s41401-023-01219-y

**Published:** 2024-01-16

**Authors:** Ling-bing Li, Ling-xiao Yang, Lei Liu, Fan-rong Liu, Alex H. Li, Yi-lin Zhu, Hao Wen, Xia Xue, Zhong-xian Tian, Hong Sun, Pei-chao Li, Xiao-gang Zhao

**Affiliations:** 1 Department of Thoracic Surgery, The Second Hospital, Cheeloo College of Medicine, Shandong University, Ji-nan, 250012 China; 2 Division of Environmental Medicine, Department of Medicine, New York University Grossman School of Medicine, New York, NY 10010 USA; 3 Department of Pharmacy, The Second Hospital, Cheeloo College of Medicine, Shandong University, Ji-nan, 250012 China; 4 Key Laboratory of Chest Cancer, The Second Hospital, Cheeloo College of Medicine, Shandong University, Ji-nan, 250012 China

**Keywords:** non-small cell lung cancer, paclitaxel resistance, triptolide, ABCB1, HNF1A, Sonic Hedgehog

## Abstract

Paclitaxel resistance is associated with a poor prognosis in non-small cell lung cancer (NSCLC) patients, and currently, there is no promising drug for paclitaxel resistance. In this study, we investigated the molecular mechanisms underlying the chemoresistance in human NSCLC-derived cell lines. We constructed paclitaxel-resistant NSCLC cell lines (A549/PR and H460/PR) by long-term exposure to paclitaxel. We found that triptolide, a diterpenoid epoxide isolated from the Chinese medicinal herb *Tripterygium wilfordii* Hook F, effectively enhanced the sensitivity of paclitaxel-resistant cells to paclitaxel by reducing ABCB1 expression in vivo and in vitro. Through high-throughput sequencing, we identified the SHH-initiated Hedgehog signaling pathway playing an important role in this process. We demonstrated that triptolide directly bound to HNF1A, one of the transcription factors of SHH, and inhibited HNF1A/SHH expression, ensuing in attenuation of Hedgehog signaling. In NSCLC tumor tissue microarrays and cancer network databases, we found a positive correlation between HNF1A and SHH expression. Our results illuminate a novel molecular mechanism through which triptolide targets and inhibits HNF1A, thereby impeding the activation of the Hedgehog signaling pathway and reducing the expression of ABCB1. This study suggests the potential clinical application of triptolide and provides promising prospects in targeting the HNF1A/SHH pathway as a therapeutic strategy for NSCLC patients with paclitaxel resistance.

**Figure:**
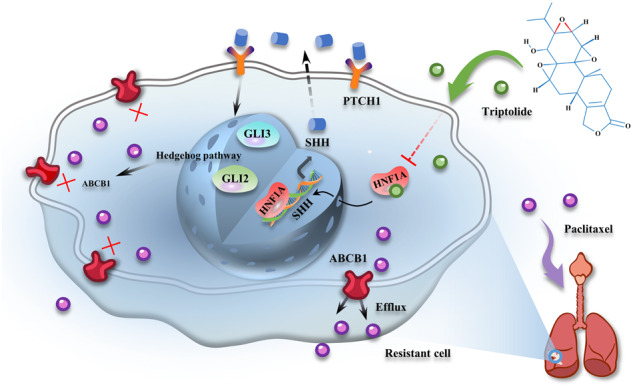
Schematic diagram showing that triptolide overcomes paclitaxel resistance by mediating inhibition of the HNF1A/SHH/ABCB1 axis.

Schematic diagram showing that triptolide overcomes paclitaxel resistance by mediating inhibition of the HNF1A/SHH/ABCB1 axis.

## Introduction

Lung cancer is one of the most common human malignancies and is the leading cause of cancer-related death for both males and females worldwide [1, 2]. Non-small cell lung cancer (NSCLC) accounts for approximately 85% of lung cancer cases and mainly includes lung squamous cell carcinoma, lung adenocarcinoma, and large cell carcinoma [3, 4]. Although great progress has been made in better understanding NSCLC biology and developing novel histopathological classification systems and promising therapeutic strategies, only 22% of NSCLC patients survive more than 5 years post-diagnosis [1]. Currently, tyrosine kinase inhibitors (TKIs) or immune checkpoint inhibitors (ICIs), in combination with chemotherapy or as a monotherapy, have been clinically applied in the therapeutic strategy for NSCLC patients with advanced staging [5, 6]. However, conventional chemotherapy remains the standard therapeutic option for most stages of NSCLC due to the low percentage of NSCLC patients suitable for TKIs or ICIs [7]. Moreover, cytotoxic chemotherapy has been recommended as a crucial therapy for NSCLC patients with relapse or metastasis after TKI or ICI therapy [8, 9]. Paclitaxel, one of the most common chemotherapy agents, has clinically benefited most NSCLC patients [7, 10]. Unfortunately, drug resistance is frequently observed among NSCLC patients with prolonged paclitaxel treatment and eventually leads to therapeutic failure and a worse outcome [11, 12]. Thus, an effective strategy for overcoming paclitaxel resistance is urgently needed in the clinical treatment of NSCLC.

A corresponding major strategy to overcome paclitaxel resistance is the repression of the ATP-binding cassette (ABC) family of transporter proteins, which can act as drug efflux pumps to excrete paclitaxel out of cells [13, 14]. Other strategies include the prevention of microtubule aggregation or microtubule breakage and regulation of apoptosis pathway-related proteins [15, 16]. ABCB1, also known as multidrug resistance protein 1 (MDR1) or P-glycoprotein, is an important member of the ABC family and confers drug resistance in cancer cells [17, 18]. In recent years, numerous traditional Chinese medicines (TCMs) have been identified as effective in reversing chemotherapy resistance by affecting ABCB1 expression [19–21]. Due to their low toxicity, minor side effects, and ability to target multiple pathways, TCMs have received increasing attention for their potential application in the treatment of cancer drug resistance. Triptolide, a diterpenoid epoxide isolated from the medicinal herb *Tripterygium wilfordii* Hook F, has been utilized for the treatment of inflammation and autoimmune diseases in Chinese medicine [22, 23]. Indeed, several studies have reported that triptolide enhances the sensitivity of various human cancers to antitumor drugs, such as doxorubicin in breast cancer [24] and cisplatin, etoposide and epirubicin in NSCLC [25]. However, the effect of triptolide in reversing paclitaxel resistance in NSCLC and the underlying molecular mechanism remains to be fully understood.

In this study, we constructed human lung cancer-derived cell lines with long-term exposure to paclitaxel as valuable tools for identifying the main factors of paclitaxel resistance. Our primary focus was to discover a TCM capable of reversing paclitaxel resistance, and to elucidate its molecular mechanisms and targets. It can provide new insights into potential clinical applications of TCM monomers or small molecule inhibitors.

## Materials and methods

### Cell lines and culture conditions

Human non-small cell lung cancer cell lines (A549, H460) were obtained from the American Type Culture Collection (ATCC, VA, USA). Cell lines were identified by using short tandem repeat (STR) analysis (Tsingke Biotechnology Co., Ltd., Beijing, China) before cell experiments. A549 and H460 cells were cultured in Roswell Park Memorial Institute (RPMI)-1640 medium (10-040-CVRC, Corning, NY, USA) with 10% fetal bovine serum (FBS) (FSP500, ExCell Bio, Shanghai, China) and 1% penicillin‒streptomycin (C125C5, New Cell & Molecular Biotech, Suzhou, China). To obtain NSCLC cell lines with acquired paclitaxel resistance (A549/PR and H460/PR), A549 and H460 cells were treated with gradually increasing concentrations (20, 40, 60, 80, 100, 120 nM) of paclitaxel (NSC125973, Selleck, Shanghai, China) by applying the method of intermittent, incremental exposure [26, 27]. To maintain paclitaxel resistance, A549/PR and H460/PR cells were cultured in a complete medium with paclitaxel (120 nM).

### Plasmids, siRNA and transfection

SHH-Flag and HNF1A-Myc plasmids and siRNAs against HNF1A, HNF1B, MYB, HOXD9, and HOXD10 were purchased from Tsingke Biotechnology (Beijing, China). Detailed information on the siRNAs is listed in Supplementary Table S1. For transient transfection, 70%–80% confluent cells were transfected with the indicated plasmids or siRNAs using Lipofectamine 3000 (L3000015, Thermo Fisher Scientific, MA, USA) or Lipofectamine RNAi MAX (13778150, Thermo Fisher Scientific, MA, USA) for 48 h according to the manufacturer’s instructions.

### Xenograft model in vivo

The athymic BALB/c nude mice (female, 4 weeks old) were purchased from Beijing Vital River Laboratory Animal Technology Co., Ltd. and maintained in a dedicated pathogen-free environment. The animal care and experimental protocols were approved by the Institutional Animal Care and Ethical Committee for Animal Experimentation of the Second Hospital of Shandong University (registration No. of the study: KYLL-2022LW118). All animal care and procedures were performed in accordance with the standards established by the Regulations on the Administration of Laboratory Animals approved by the State Council of the People’s Republic of China. The A549 and A549/PR cells were collected and diluted to a 5 × 10^7^ cells/mL cell suspension. A total of 100 μL of suspended cells was subcutaneously injected into the armpit of each nude mice. One week after the injection of tumor cells, the tumor size was evaluated by electronic calipers every 3 days and calculated following the formula length × width^2^ × 1/2. Meanwhile, each group was intraperitoneally administered triptolide (0.1 mg/kg, per 3 days) and/or paclitaxel (5 mg/kg, per 6 days). The mice were sacrificed after 24 days of treatment, and tumor tissues were excised and fixed in 4% paraformaldehyde solution for further study.

### Clinical samples and tissue microarray construction

From 2017 to 2020, 90 cases of NSCLC from surgical patients in our department were retrospectively reviewed. The Ethics Committee of Second Hospital of Shandong University approved and supervised our protocol, and all patients were informed before surgery (registration No. of the study: KYLL-2022LW116). None of these patients received any preoperative chemotherapy or radiotherapy. Patients were staged according to the tumor‐node‐metastasis (TNM) classification from the 8th Union for International Cancer Control (UICC).

NSCLC tissue microarrays (TMAs) were created in the Laboratory of Thoracic Cancer, Shandong University, using a fully automated TMA Grand Master (3D Histech, Budapest, Hungary). The H&E slides of NSCLC tumor tissue samples and adjacent normal tissue samples were reviewed by an attending pathologist (Dr Bing-Xin Guan and Dr Yu-Peng Deng, Second Hospital of Shandong University) for selection of the most representative donor blocks. The region to be punched for the TMA construct was circled and matched to the corresponding donor blocks. After eliminating damaged samples, the remaining 78 analyzable samples were analyzed for immunohistochemical staining. The detailed clinical and pathological features of the patients are shown in Table 1.

**Table 1:** Clinicopathological correlation of HNF1A and SHH expression in NSCLC.

	Cases (*n* = 78)	HNF1A expression			SHH expression		
		Weak (*n* = 35)	Strong (*n* = 43)	*P* value	Weak (*n* = 41)	Strong (*n* = 37)	*P* value
Gender							
Male	42	18	24	0.6992	21	21	0.6242
Female	36	17	19		20	16	
Age (years)							
<60	31	14	17	0.9667	17	14	0.7439
≥60	47	21	26		24	23	
Clinical stage							
I	53	31	22	**0.0004**	33	20	**0.0125**
II + III	25	4	21		8	17	
T stage							
T1 + T2	63	33	30	**0.0063**	35	28	0.2782
T3 + T4	15	2	13		6	9	
N stage							
N0	65	34	31	**0.0032**	38	27	**0.0197**
N1 + N2	13	1	12		3	10	

The *χ*^2^ analysis of the clinicopathological characteristics between clusters.

Statistical significance (*P* < 0.05) is shown in bold.

### Immunohistochemistry (IHC)

Tumor tissues were immediately formalin-fixed and paraffin-embedded after surgical resection for IHC. The processed tissues were subsequently embedded in paraffin, cut into 0.4 μm sections, and mounted onto microscope glass slides. Then, the sections underwent dewaxing, rehydration, antigen retrieval, and endogenous peroxidase blocking. Goat serum was used to block nonspecific binding on sections at room temperature for an hour. Sections were incubated in primary antibody overnight at 4 °C, followed by incubation with secondary antibody conjugated by horseradish peroxidase for an hour at room temperature. The signals were developed by DAB solution (PV-9001, ZSGB-BIO, Beijing, China) and counterstained with hematoxylin.

### Cell viability assays

The Cell Counting Kit-8 (C0005, TargetMol, Shanghai, China) assay was used to evaluate the proliferation of the cells. Cells were seeded in 96-well plates at 4000 cells per well. After treatment with the indicated concentrations of paclitaxel for 48 h and/or triptolide (NSC125973, Selleck, Shanghai, China) for 24 h, 10 μL of CCK-8 solution was added to each well and incubated at 37 °C for 2 h. The number of viable cells was measured according to the absorbance at 450 nm, which was detected using a microplate reader (M200 PRO, NanoQuant, Männedorf, Switzerland).

### Immunofluorescence

Cells were plated in a glass-bottom cell culture dish (BS-15-GJM, Biosharp, Hefei, China) and fixed in 4% paraformaldehyde at room temperature for 15 min when the cell confluence reached 60%–80%, and then the cells were permeabilized with 0.3% Triton X-100 for 20 min. Next, the cells were blocked with PBS containing 5% goat serum for 1 h at room temperature, followed by incubation with the indicated primary antibodies (Supplementary Table S2) overnight at 4 °C. After washing with PBS, the cells were incubated with goat anti-rabbit IgG conjugated with CoraLite488 (1:500) (SA00013-2, Proteintech, Wuhan, China) in the dark for an hour and then incubated with antifade mounting medium with DAPI (P0131, Beyotime, Shanghai, China) for 10 min. The signals from targeted proteins were visualized using a confocal laser scanning microscope (LSM 800, Zeiss, Oberkochen, Germany).

### Enzyme-linked immunosorbent assay (ELISA)

The supernatant was collected from the experimental and control groups in 6-well plates when the cell confluence was approximately 90%. ELISA was performed to detect the abundance of SHH protein according to the instructions (ELH-ShhN, RayBiotech, GA, USA).

### Reverse transcription and quantitative polymerase chain reaction (RT‒qPCR)

Total RNA was isolated from cultured cell lines by the Total RNA Kit (DP419, TIANGEN, Beijing, China) following the manufacturer’s protocols. Reverse transcription was performed using the cDNA First Strand Kit (KR116, TIANGEN, Beijing, China). Real-time quantitative PCR analysis of cDNA was performed using the SYBR Green Fluorescence Quantification Kit (FP402, TIANGEN, Beijing, China) on a QuantStudio^TM^ 5 (Thermo Scientific, MA, USA) instrument. GAPDH was used as an internal control gene. Relative expression was calculated using the 2^–ΔΔCt^ normalization method. The primers used for this study are listed in Supplementary Table S3.

### Western blotting (WB)

Total protein was extracted from cells by using boiling buffer [10 mM Tris-HCl (pH 7.4), 1 mM Na_3_VO_4_, and 1% SDS]. Nucleoprotein was extracted using a cytoplasmic and nuclear extraction kit (SC-003, Invent Biotechnologies, MN, USA). The proteins were separated using sodium dodecyl sulfate‒polyacrylamide gel electrophoresis (SDS‒PAGE) and transferred to nitrocellulose membranes (HATF00010, Merck Millipore, Germany). The membranes were blocked in Tris-buffered saline (TBS) supplemented with 5% nonfat milk at room temperature for an hour before overnight hybridization with appropriate concentrations of primary antibodies (Supplementary Table S2). The membrane was then incubated with the corresponding secondary antibody for 1 h at room temperature. An enhanced chemiluminescent substrate (AR1197, Boster, Wuhan, China) was used to detect the signal of targeted proteins on a Tanon Imaging System.

### Chromatin immunoprecipitation (ChIP)

A ChIP assay was performed using a ChIP Assay Kit (P2078, Beyotime, Shanghai, China) according to the manufacturer’s instructions. In brief, nuclear lysates were sonicated to shear DNA to approximately 200–300 bp, followed by immunoprecipitation overnight at 4 °C using rabbit IgG or an antibody against HNF1A (Supplementary Table S2). Immunoprecipitated DNA was purified by using a universal DNA purification kit (DP214, Tiangen, Beijing, China) and then analyzed by real-time PCR. The relative expression level of the target gene was normalized to the adjusted input [CT input – log2 (starting input fraction)] and calculated using the 2^–ΔΔCT^ method. The primer sequences used in the ChIP assay are listed in Supplementary Table S3.

### Dual-luciferase reporter assay

The pRL-TK and pGL3-Basic plasmids were purchased from Tsingke Biotechnology (Beijing, China). The SHH promoter sequence (from −499 to 101 bp referring to the transcriptional start site) containing the wild-type HNF1A motif (SHH-pGL3) or mutated HNF1A motif (SHH-Mut#1-pGL3, SHH-Mut#2-pGL3, and SHH-Mut#3-pGL3) was inserted between the *Kpn* I and *Xho* I sites into a pGL3-Basic plasmid to regulate the expression of firefly luciferase. Different groups of cells were transiently cotransfected with phRL-TK in combination with SHH-pGL3, SHH-Mut#1-pGL3, SHH-Mut#2-pGL3, or SHH-Mut#3-pGL3 plasmids by using Lipofectamine 3000 (L3000015, Thermo Fisher Scientific, MA, USA). After 48 h of transfection, the luciferase activity was measured using a Dual-Luciferase Reporter Assay System (E1960, Promega, WI, USA) in a microplate luminometer (Cytation5, Biotek, VT, USA). The luminescent signal from the renilla reaction was used as an internal control.

### RNA sequencing and data analysis

Total RNA was isolated and purified using TRIzol reagent (Invitrogen, CA, USA) following the manufacturer’s procedure. RNA samples were sequenced on an Illumina NovaSeq™ 6000 following the vendor’s recommended protocol (Lianchuan BioTechnology, Hangzhou, China) [28]. Bioinformatics analysis was then performed on the company’s website (https://www.omicstudio.cn/home), and the downloaded data were graphed using the R package.

### TCGA and GEO data

TCGA NSCLC expression data were downloaded from The Cancer Genome Atlas (TCGA) data portal (https://www.cancer.gov/ccg/research/genome-sequencing/tcga). We downloaded relevant quantification matrices from the NCBI GEO database (https://www.ncbi.nlm.nih.gov/geo/, GEO accession numbers GSE10245 and GSE77803) in MINiML format. Box line plots, PCA plots and correlation analysis were drawn by the R package.

### Molecular docking

The HNF1A protein structure was obtained from the AlphaFold database, while the chemical structure of Triptolide was downloaded from the PubChem database. The protein and ligand files were prepared by converting all molecular files to PDBQT format, removing water molecules, and adding polar hydrogen atoms. Molecular docking analyses were conducted using Autodock Vina software to visualize the models.

### Isothermal titration calorimetry (ITC)

Isothermal titration calorimetry (ITC) was conducted on MicroCal PEAQ-ITC (Malvern, Worcestershire, UK). Recombinant HNF1A protein (40 μM) and triptolide (400 μM) were dissolved in TBS buffer, respectively. The solution was then centrifuged (12,000 rpm) for 10 min at 4 °C and the supernatant was collected. The experiment was run following the manufacturer’s instructions using the following parameters: sample pool volume, 200 μL; injection needle volume, 40 μL; temperature was set to 25 °C; 19 total injections. Based on the measured isotherms, the following thermodynamic parameters were calculated by curve fitting: binding stoichiometry (N); binding constant (KD); enthalpy (ΔH); entropy (ΔS).

### Statistical analysis

All data were statistically analyzed using GraphPad Prism 8.0 (GraphPad Software, CA, USA). All experiments were repeated at least three times in this study. All data are presented as the means ± standard deviations (SD) unless otherwise indicated. The Kaplan–Meier method was used to generate survival curves, and the statistical significance of the difference in survival between the two groups was determined by the log-rank test. Correlation analysis was performed with Pearson’s correlation. The *χ*^2^ analysis was used to evaluate the correlation between the average optical density of the indicated protein and clinicopathological characteristics. *P* values < 0.05 were considered to indicate statistical significance.

## Results

### Establishment of paclitaxel-resistant NSCLC cell lines

**Fig. 1:**
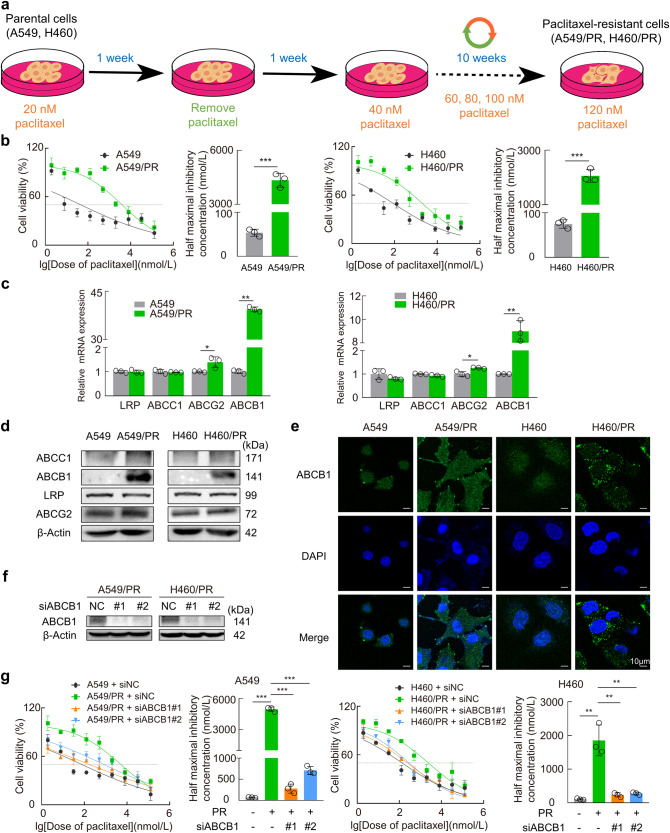
Establishment of paclitaxel-resistant cell lines (A549/PR and H460/PR). **a** Diagrams show the treatment of parental A549 and H460 cells with gradients of paclitaxel to produce A549/PR and H460/PR cells. **b** Cell viability was detected by the CCK-8 method, and the dose–response curves of paclitaxel in parental and resistant cells were plotted. The IC_50_ value was calculated according to the fitting curves. **c**, **d** Results of RT‒qPCR and Western blotting of LRP, ABCB1, ABCC1, and ABCG2 in parental and paclitaxel-resistant cells. **e** Immunofluorescence staining detected ABCB1 expression in the parental and resistant groups. **f** Protein expression was assessed using a Western blot assay following knockdown of ABCB1. **g** The cell viability was assessed, and a dose–response curve of paclitaxel in ABCB1 knockdown resistant cells was constructed. The IC_50_ values were determined based on the fitted curves. Data are presented as the mean ± SD, *P < 0.05, **P < 0.01, ***P < 0.001. PR paclitaxel resistance.

To generate paclitaxel-resistant NSCLC cell lines, we treated parental A549 and H460 cells with paclitaxel starting at a low dose with intermittent incremental increases (20, 40, 60, 80, 100, 120 nM) according to the strategy shown in Fig. 1a. Approximately 10 weeks later, the half-maximal inhibitory concentration (IC_50_) values of paclitaxel were assessed by measuring the percent inhibition of the parental and resistant cells at 48 h. The IC_50_ values of paclitaxel in the parental cell lines of A549 and H460 were 54 ± 4.533 and 76.57 ± 4.057 nM, respectively. However, the IC_50_ values in A549/PR and H460/PR cells were 4339 ± 224.5 and 2060 ± 137.3 nM, respectively (Fig. 1b). The IC_50_ of paclitaxel was much higher in both resistant cell lines than in the corresponding parental cells. Here, although we verified that A549/PR and H460/PR cells remained drug resistant after passaging and transfection, each experiment required parental cells as a control to quantify the sensitivity of paclitaxel-resistant cells to the drugs.

Some studies have suggested that upregulation of ABC family proteins such as ABCB1, ABCC1 and ABCG2, which transport chemotherapeutic drugs out of cancer cells, is the main reason for chemoresistance in cancer cells [29, 30]. For this reason, we then detected the mRNA and protein expression levels of resistance indicators such as lung resistance-related protein (LRP), ABCB1, ABCC1 and ABCG2 in NSCLC paclitaxel-resistant cell lines. The findings demonstrated that among these factors, ABCB1 exhibited the most significant upregulation in paclitaxel-resistant cells at both mRNA and protein levels (Fig. 1c, d). Confocal immunofluorescence results showed that ABCB1 expression was significantly elevated in both the membrane and cytoplasm of paclitaxel-resistant cells (Fig. 1e). Furthermore, the knockdown of ABCB1 in both paclitaxel-resistant cells was observed to lead to a significant reduction in the IC_50_ value of paclitaxel (Fig. 1f, g). These results demonstrated the successful establishment of paclitaxel-resistant cell sublines and confirmed that the heightened expression of ABCB1 constituted an important factor in the emergence of resistance to paclitaxel in NSCLC cells.

### Triptolide significantly reverses ABCB1-mediated NSCLC paclitaxel resistance in vitro and in vivo

**Fig. 2:**
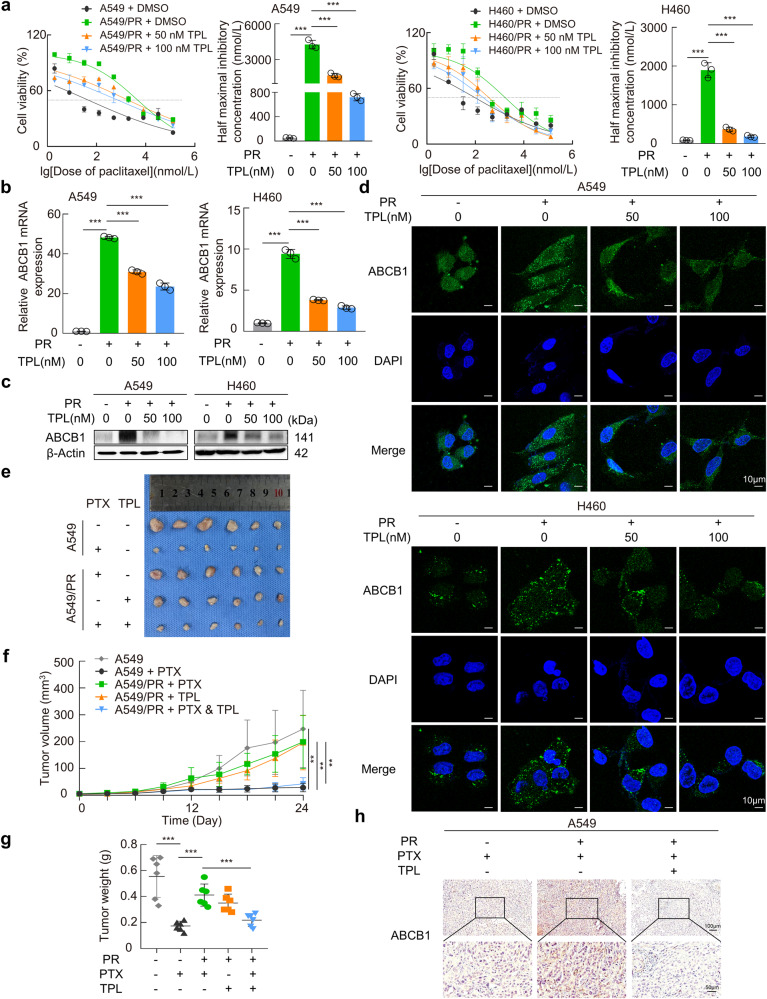
Triptolide reverses paclitaxel resistance in NSCLC via downregulation of ABCB1. **a** Plotting and evaluation of the dose–response curves of paclitaxel in cells after 24 h of triptolide cotreatment. The IC_50_ of paclitaxel after combined triptolide treatment was calculated based on the fitted curves. **b**, **c** ABCB1 expression levels were evaluated by RT‒qPCR and Western blotting in different treatment groups. **d** Confocal immunofluorescence staining showed changes in ABCB1 protein around the time of triptolide treatment. **e** Subcutaneous xenograft tumors derived from parental and resistant cells under different treatments. **f** Growth curve of subcutaneous xenograft tumors. **g** Tumor weight of subcutaneous xenografts. **h** IHC staining was applied to detect ABCB1 protein levels in tumor sections from xenograft models of different treatment groups. Data are presented as the mean ± SD, **P < 0.01, ***P < 0.001. TPL triptolide, PR paclitaxel resistance.

In previous studies, triptolide sensitized cancer cells to a variety of therapies, including chemotherapy, cytokine therapy, and radiotherapy, and showed no significant adverse effects in xenograft mice models [31]. We treated paclitaxel-resistant cells with varying concentrations of triptolide (25, 50, 100, and 150 nM) and observed that while triptolide at concentrations of 25, 50, and 100 nM had no discernible impact on the proliferative viability of paclitaxel-resistant cells, a concentration of 150 nM significantly inhibited the proliferation of A549/PR and H460/PR cells (Supplementary Fig. S1a). Strikingly, by determining the IC_50_ values of paclitaxel in resistant cells treated with 50 and 100 nM of triptolide, the results showed that triptolide could significantly reduce the resistance phenotype of resistant cells to paclitaxel (Fig. 2a). These results suggest that it is not the case that triptolide reversed the resistance of paclitaxel-resistant cells to paclitaxel by affecting cell proliferation viability. Rather, these suggested that triptolide may affect the sensitivity of paclitaxel-resistant cells to paclitaxel through a potential mechanism. In addition, triptolide dose-dependently decreased ABCB1 mRNA and protein expression in A549/PR and H460/PR cells and reduced ABCB1 immunofluorescence intensity on the cell membrane and in the cytoplasm (Fig. 2b–d). To further verify the effect of triptolide on reversing paclitaxel resistance in vivo, BALB/c nude mice were subcutaneously xenografted with A549 or A549/PR cells followed by treatment with paclitaxel and/or triptolide. Monotherapy with paclitaxel significantly suppressed the growth of subcutaneous tumors derived from A549 cells to a greater degree than that of tumors derived from A549/PR cells. Compared to paclitaxel or triptolide monotherapy, the combination markedly inhibited the growth of subcutaneous tumors in the A549/PR group (Fig. 2e–g). The administration of paclitaxel and/or triptolide did not result in significant weight reduction in mice (Supplementary Fig. S1b). The hepatotoxicity and nephrotoxicity induced by treatment with paclitaxel and/or triptolide are presented in Supplementary Tables S4 and S5, respectively. The results revealed a significant elevation in serum AST activity within the triptolide treatment group. Both AST and ALT activities were significantly higher in the paclitaxel group. The combined administration of triptolide and paclitaxel resulted in a more pronounced increase in ALT and AST activity. No significant differences were observed in other hepatic and renal parameters. Further, consistent with triptolide-induced suppression of ABCB1 in vitro, IHC revealed a significant increase in ABCB1 protein levels in tumor sections from A549/PR xenograft mice compared with A549 xenograft mice, while triptolide treatment dramatically decreased the protein level of ABCB1 in the A549/PR group (Fig. 2h). Collectively, our results demonstrated that triptolide was able to override paclitaxel resistance in NSCLC cells by regulating ABCB1 expression.

### Triptolide reverses paclitaxel resistance via blocking the Hedgehog pathway

**Fig. 3:**
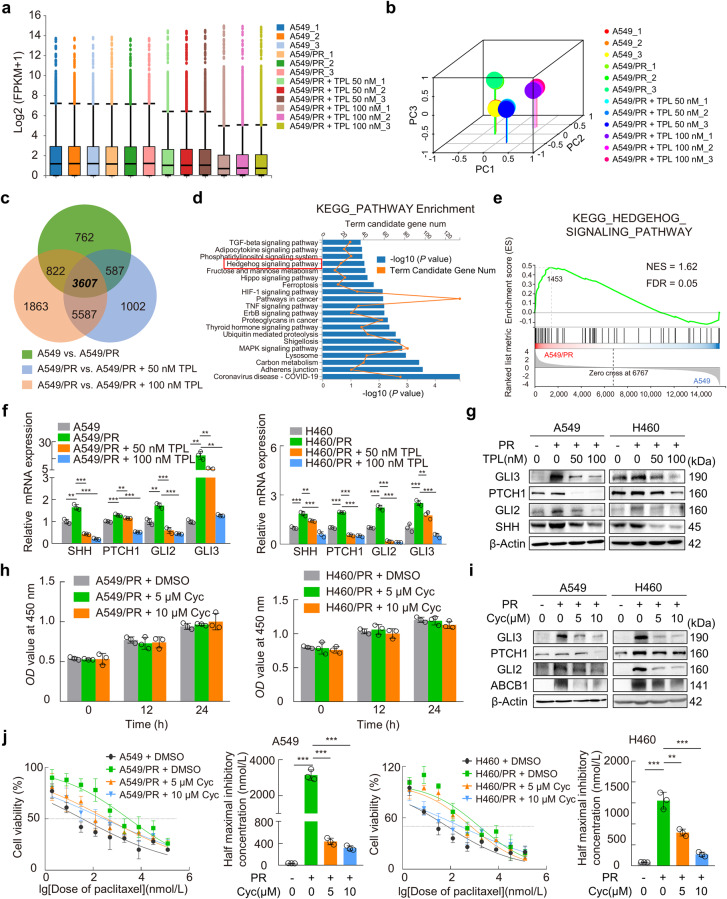
Triptolide reverses cellular resistance to paclitaxel by inhibiting the Hedgehog pathway. **a** Box plots showing the distribution of gene expression levels for each group in the sequencing data. **b** 3D-plot of principal component analysis (PCA) analysis of different treatment groups. **c** Overlapping DEGs from the three comparisons were shown in the Venn diagram according to the RNA-seq data. **d** Representative terms from KEGG pathway enrichment analysis based on the overlapping DEGs. **e** GSEA was performed in KEGG Hedgehog signaling pathway gene sets. **f**, **g** RT‒qPCR and Western blotting verified the expression levels of SHH, PTCH1, GLI2, and GLI3 in the indicated cells with or without triptolide treatment. **h** A549/PR and H460/PR cells were treated with cyclopamine (5 or 10 μM) for 24 h. A CCK-8 assay was applied to quantify cell viability. **i** Western blotting was used to detect the protein levels of the indicated genes in cyclopamine-treated paclitaxel-resistant cells. **j** Dose–response curves of paclitaxel in cyclopamine-treated resistant cells were plotted based on CCK-8 assays. Histograms revealed the IC_50_ value calculated from the fitted curves. Data are indicated as the mean ± SD, **P < 0.01, ***P < 0.001. TPL triptolide, Cyc cyclopamine, PR paclitaxel resistance.

Subsequently, we performed RNA-Seq analysis to elucidate the mechanism underlying the reversal of paclitaxel resistance by triptolide. After analysis of all coding genes in the sequencing data, the distribution of expression of these genes in each treatment group was determined (Fig. 3a). In addition, three-dimensional principal component analysis (PCA) showed that the clustered samples were aligned with the actual treatment groupings (Fig. 3b). The profiles of differentially expressed genes (DEGs) were then obtained by grouping as shown in Fig. 3c; 3607 DEGs were commonly shared among the groups. Here, a pathway analysis based on the KEGG database was performed for DEGs. It was reported that the Hedgehog signaling pathway promotes the chemotherapy resistance of tumors by regulating ABCB1 expression [32, 33]. Therefore, the Hedgehog signaling pathway caught our interest and attention (Fig. 3d). Consistently, GSEA revealed the upregulation of the Hedgehog signaling pathway in the A549/PR group compared to the A549 or triptolide-treated groups (Fig. 3e and Supplementary Fig. S2a). Intriguingly, out of three Hedgehog ligands (SHH, DHH, and IHH), only SHH (and its downstream genes PTCH1, GLI2, and GLI3) exhibited upregulation in A549/PR cells compared to A549 cells, but these factors were downregulated upon treatment with triptolide according to RNA-seq data (Supplementary Fig. S2b). Consistent with the RNA-seq results, the mRNA and protein levels of SHH, PTCH1, GLI2, and GLI3 were significantly upregulated in paclitaxel-resistant cells compared with their parental cells, but their levels were drastically reduced in triptolide-treated cells in a dose-dependent manner (Fig. 3f, g). When CHX impeded the synthesis of GLI2 and GLI3 proteins, triptolide (50 nM) exhibited a reduction in the levels of these proteins, indicating their diminished stability (Supplementary Fig. S2c).

To determine the function of the Hedgehog pathway in paclitaxel-resistant NSCLC cells, we treated resistant cells with 5 or 10 μM cyclopamine, an inhibitor of the Hedgehog signaling pathway, for 24 h (no significant effect on cell viability, Fig. 3h). The results in Fig. 3i, j show that cyclopamine markedly decreased the protein expression levels of PTCH1, GLI2, GLI3, and ABCB1 and decreased the IC_50_ values of paclitaxel in resistant cells. These results suggested that triptolide could effectively overcome ABCB1-mediated paclitaxel resistance by inhibiting the Hedgehog signaling pathway.

### Restricted initiation of SHH expression is crucial for triptolide to overcome paclitaxel resistance

**Fig. 4:**
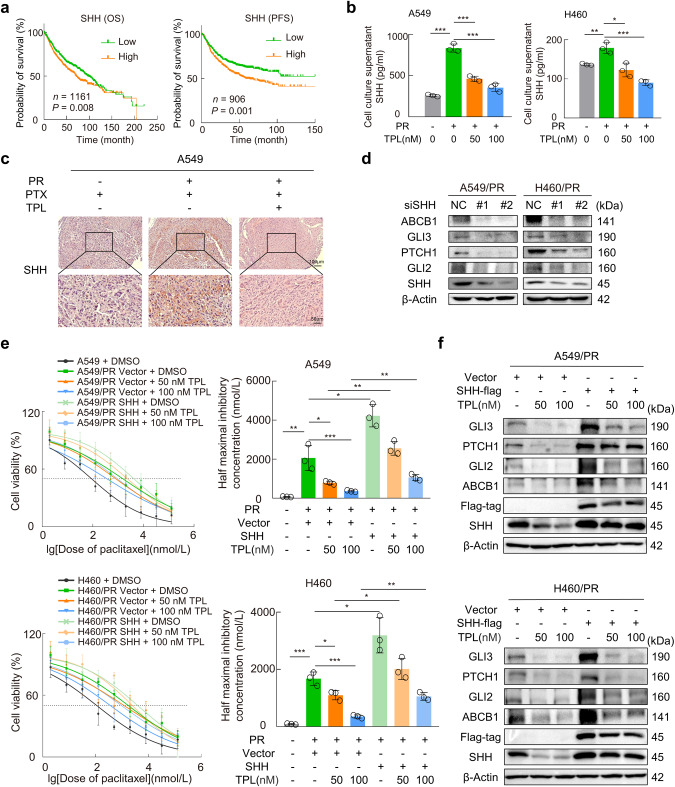
SHH plays a crucial role in the triptolide-induced reversal of paclitaxel resistance. **a** Kaplan–Meier plot of LUAD patient OS and PFS stratified by SHH expression status. **b** ELISA was used to detect the concentration of SHH in the cell supernatants of different groups. **c** Representative images of SHH immunohistochemical staining from tumor slides in different treatment groups of mice. **d** Western blotting was used to detect the expression levels of the indicated proteins after SHH knockdown. **e** Dose–response curves of paclitaxel in SHH-overexpressing A549/PR cells with or without triptolide were determined. The histogram showed the estimated IC_50_ values based on the fitting curves. **f** A549/PR or H460/PR cells were transfected with SHH-overexpressing plasmids followed by triptolide treatments for 24 h. Western blotting was applied to detect the expression levels of the indicated proteins. Data are indicated as the mean ± SD, *P < 0.05, **P < 0.01, ***P < 0.001. TPL triptolide, PR paclitaxel resistance, OS overall survival, PFS progression-free survival.

To explore how triptolide inhibits the Hedgehog pathway in paclitaxel-resistant cells, we focused on SHH, the upstream initiator of Hedgehog signaling. Overall survival (OS) is defined as the time from randomization to death (from any cause) [34]. Progression-free survival (PFS) is the time from randomization or initiation of treatment to disease progression or death [35]. Kaplan–Meier plotter (https://kmplot.com/analysis/) showed that high SHH mRNA expression was associated with poor OS and PFS (Fig. 4a) [36]. The results showed that triptolide decreased the mRNA and protein expression of SHH in paclitaxel-resistant cells (Fig. 3f, g). Moreover, we examined the concentration of SHH ligand, a secreted protein that binds to the transmembrane receptor PTCH1, in the supernatant of resistant cells. ELISA showed that SHH secretion was significantly reduced in the supernatant of paclitaxel-resistant cells treated with triptolide (Fig. 4b). In the xenograft model, upregulation of SHH protein in the A549/PR group was inhibited by triptolide treatment (Fig. 4c). The subsequent discovery unveiled a remarkable downregulation of Hedgehog downstream targets and ABCB1 expression upon the knockdown of SHH in paclitaxel-resistant cells (Fig. 4d). The IC_50_ curve fitting results obtained from the cell viability assay demonstrated a significant reduction in paclitaxel resistance in SHH knockdown cells (Supplementary Fig. S3a). In addition, cell viability assays showed that SHH overexpression attenuated the decrease in the IC_50_ value of paclitaxel induced by triptolide in resistant cells (Fig. 4e). Moreover, ectopic expression of SHH significantly increased the protein expression levels of PTCH1, GLI2, GLI3, and ABCB1 in paclitaxel-resistant cells treated with triptolide (Fig. 4f). These results supported that triptolide reversed ABCB1-mediated paclitaxel resistance by downregulating SHH in NSCLC cells.

### HNF1A triggers initiation of SHH as a transcription factor

**Fig. 5:**
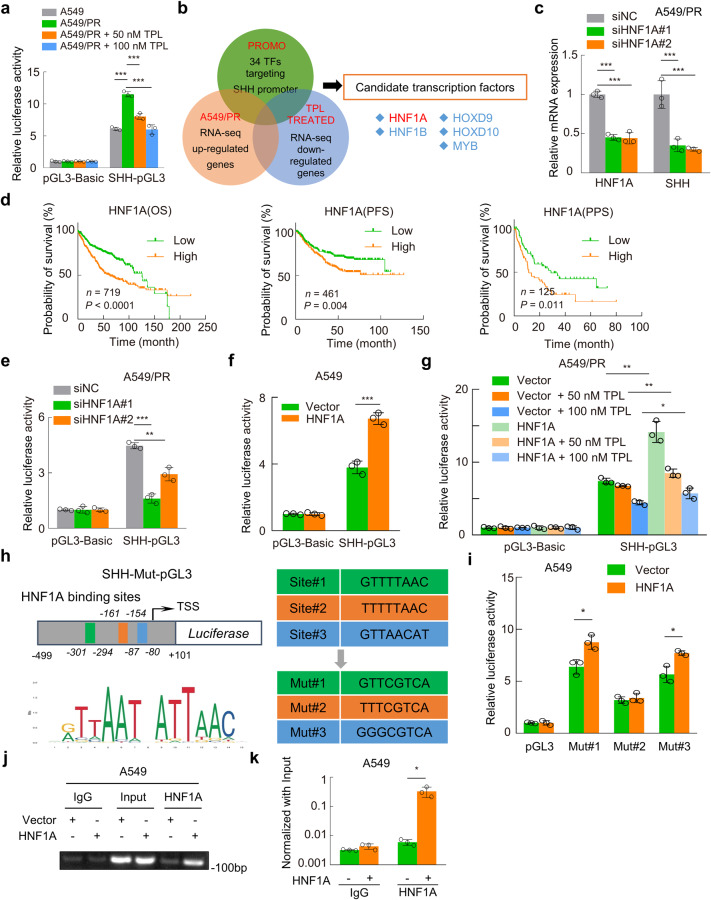
HNF1A acts as a transcription factor that binds directly to the SHH promoter region. **a** The dual-luciferase reporter assay was used to detect the luciferase activity of different groups after transfection for 48 h. **b** Venn diagram showing five shared genes among the three indicated groups. **c** RT‒qPCR was performed to detect the expression levels of HNF1A and SHH mRNA in different groups. **d** Kaplan–Meier curves showing the OS, PFS, and PPS among LUAD patients in the TCGA database stratified by HNF1A expression. **e**, **f** Detection of SHH-pGL3 promoter luciferase activity in HNF1A-knockdown A549/PR cells and HNF1A-overexpressing A549 cells. **g** Luciferase activity was measured at 48 h post transfection and 24 h after triptolide treatment. **h** The schematic diagram showed the promoter region of SHH (−499 to +101 bp), and motif analysis of the HNF1A binding element was performed. Mutant (Mut) promoter sequences were designed for insertion into the luciferase vector (pGL3-Basic) based on the predicted binding site sequence. **i** Detection of mutant promoter activity in HNF1A-overexpressing A549 cells using dual-luciferase reporter assays. **j** ChIP assays were performed using a specific HNF1A or IgG antibody. The results were semi-quantified by agarose gel electrophoresis. **k** Quantitative analysis of ChIP results using real-time qPCR. Data are presented as the mean ± SD, *P < 0.05, **P < 0.01, ***P < 0.001. TPL triptolide, OS overall survival, PFS progression-free survival, PPS post-progression survival.

To elucidate the mechanism underlying SHH transcriptional inhibition induced by triptolide, we evaluated the transcriptional activity of the SHH promoter using a dual-luciferase reporter assay and found that SHH transcriptional activity was enhanced in A549/PR cells compared with A549 cells. However, the increased activity of the SHH promoter in A549/PR cells was markedly inhibited by triptolide treatment, and the effect was dose-dependent (Fig. 5a); SHH promoter activity and SHH expression level showed consistent changes upon triptolide treatment.

Next, the PROMO website was used to predict the possible transcription factors (TFs) binding to the SHH promoter region [37]. Among 34 TFs predicted by PROMO, five TFs (HNF1A, HNF1B, HOXD9, HOXD10, and MYB) were upregulated in A549/PR cells compared with A549 cells and downregulated in a dose-dependent manner in triptolide-treated A549/PR cells (Fig. 5b). After RT-qPCR validation, it was found that the expression of all five transcription factors was increased in both A549/PR and H460/PR paclitaxel-resistant cells relative to the parental cells, and decreased dose-dependently after triptolide treatment (Supplementary Fig. S4a). Exploratively, we interfered with the expression of each of the five candidate TFs in A549/PR cells with specific siRNAs and found that knockdown of HNF1A, HNF1B, or HOXD10 expression significantly downregulated SHH mRNA levels (Fig. 5c and Supplementary Fig. S4b). Post-progression survival (PPS) is defined as the time from date of progression to death [38]. We analyzed the effects of five candidate TFs on OS, PFS, and PPS by using the website tool Kaplan–Meier Plotter [36]. Only high HNF1A expression was associated with poor OS, PFS, and PPS among LUAD patients (Fig. 5d and Supplementary Fig. S5a).

Then, a dual-luciferase reporter assay revealed that knockdown of HNF1A inhibited SHH promoter activity in A549/PR cells, while ectopic HNF1A expression increased the activity of the SHH promoter in A549 cells (Fig. 5e, f). Interestingly, overexpression of HNF1A attenuated triptolide-induced inhibition of SHH transcription in A549/PR cells (Fig. 5g). Moreover, three predicted binding sites from the PROMO website were mutated (Fig. 5h) to confirm the exact binding sequence on the SHH promoter region for HNF1A. Notably, dual-luciferase reporter assays showed that only mut#2 exhibited no elevated SHH promoter activity in A549/PR cells (Fig. 5i). It indicated that the TTTTTAAC region located at −161 to −154 bp of the SHH promoter is required for HNF1A-mediated SHH transcription. We then designed primers (Supplementary Table S3) covering the TTTTTAAC region of the SHH promoter and confirmed by ChIP-qPCR analysis that HNF1A protein can bind directly to this region (Fig. 5j, k). In addition, subsequent ChIP-qPCR experiments revealed that triptolide effectively attenuated the binding ability of HNF1A protein to the SHH promoter region (Supplementary Fig. S5b, c). Overall, we identified a novel SHH transcription factor, HNF1A, utilizing bioinformatic analysis and experiments in paclitaxel-resistant cells.

### HNF1A modulates the Hedgehog pathway and ABCB1 expression via SHH

**Fig. 6:**
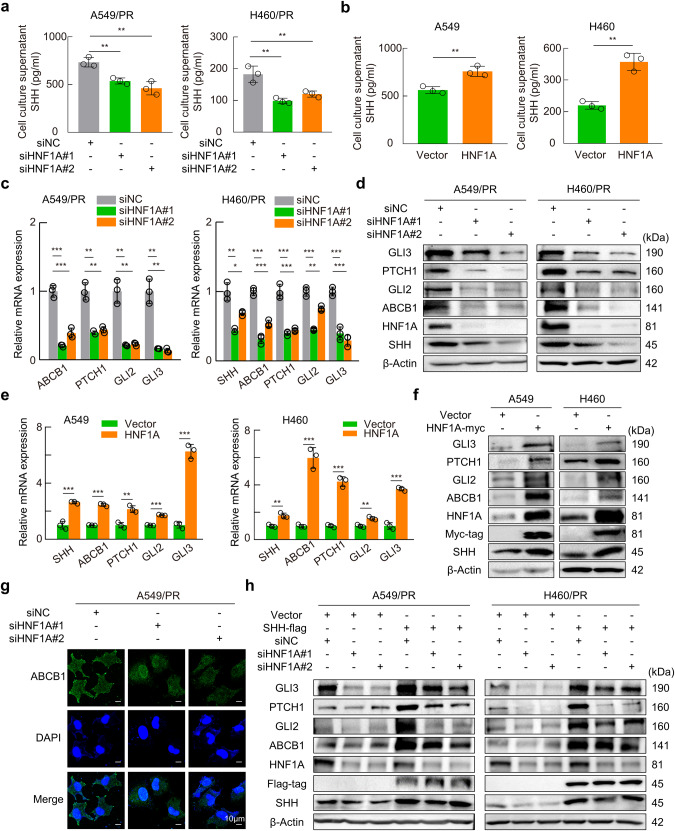
HNF1A regulates the SHH signaling pathway and ABCB1 expression. **a**, **b** ELISAs were used to detect the concentration of SHH in the supernatant of HNF1A-knockdown and -overexpression cells. **c**, **d** RT‒qPCR and Western blotting were used to detect the mRNA and protein levels of the indicated genes after HNF1A knockdown. **e**, **f** Total RNA and protein were isolated from cells transfected with HNF1A-expressing plasmid. RT‒qPCR and Western blotting were used to evaluate the expression levels of the indicated genes. **g** Immunofluorescence was used to assess the location and abundance of ABCB1 protein in HNF1A-knockdown A549/PR cells. **h** Western blot analysis of the effect of HNF1A knockdown on the Hedgehog pathway and ABCB1 protein expression in SHH-overexpressing cells. Data are presented as the mean ± SD, *P < 0.05, **P < 0.01, ***P < 0.001. TPL triptolide, PR paclitaxel resistance.

To investigate whether SHH and its downstream pathways were regulated by HNF1A, we first examined the effect of HNF1A on SHH secretion. ELISAs revealed that knockdown of HNF1A in paclitaxel-resistant cells significantly reduced the concentration of secreted SHH protein. In contrast, overexpression of HNF1A in parental cells was associated with a significant increase in SHH secretion (Fig. 6a, b). In addition, knocking down HNF1A drastically reduced the mRNA and protein expression levels of Hedgehog pathway components and ABCB1 in paclitaxel-resistant cells (Figs. 5c and 6c, d). Meanwhile, overexpression of HNF1A in parental cells resulted in a significant upregulation of SHH and its downstream mRNAs and proteins (Fig. 6e, f). Furthermore, we transfected HNF1A-knockdown A549/PR cells and HNF1A-overexpressing A549 cells with 8× GliBS luciferase, a GLI protein-dependent reporter gene utilized for Hedgehog signaling analysis. The results demonstrated that knockdown of HNF1A in paclitaxel-resistant cells significantly attenuated the activation of Hedgehog signaling, whereas overexpression of HNF1A in parental cells markedly enhanced the activation of Hedgehog signaling (Supplementary Fig. S6a).

A cell viability assay demonstrated that IC_50_ values were clearly decreased in paclitaxel-resistant cells with HNF1A-knockdown compared with control cells (Supplementary Fig. S6b), which was further confirmed by decreased immunofluorescence intensity of ABCB1 in paclitaxel-resistant cells with HNF1A knockdown (Fig. 6g and Supplementary Fig. S6c). The knockdown of SHH in paclitaxel-resistant cells overexpressing HNF1A, resulted in the abolishment of HNF1A’s ability to effectively upregulate ABCB1 protein levels (Supplementary Fig. S6d). Moreover, overexpressing SHH significantly restored the downregulation of PTCH1, GLI2, GLI3, and ABCB1 in paclitaxel-resistant cells with HNF1A knockdown (Fig. 6h). These results confirmed that HNF1A regulates the expression of Hedgehog pathway components and ABCB1 by mediating SHH.

### HNF1A is the crucial target by which triptolide reverses paclitaxel resistance

**Fig. 7:**
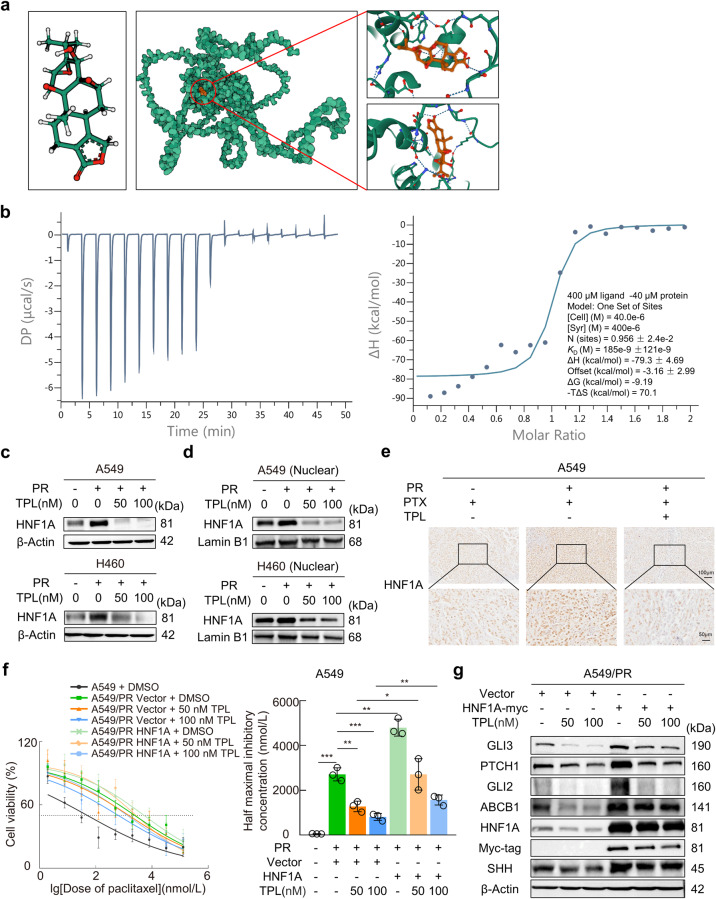
Targeted inhibition of HNF1A by triptolide reverses paclitaxel resistance. **a** Chemical structure of triptolide (left), binding mode of triptolide to HNF1A (middle), 3D binding structures of triptolide and HNF1A magnified from various angles (right). White is hydrogen, green is carbon, red is oxygen, and blue is nitrogen. The dotted lines indicated the forces of interaction of the small molecules with the protein structure. **b** Isothermal titration calorimetry revealed the binding of HNF1A ligand-binding domain protein to triptolide. **c** Western blotting indicated HNF1A expression levels in each treatment group. **d** Western blotting was performed to detect the protein expression of HNF1A in the nucleus. Lamin B1 was used as a control. **e** Immunohistochemical staining was applied to assess the location and expression level of HNF1A in tumor sections of xenograft models from different treatment groups. **f** CCK-8 cell viability assays revealed dose–response curves for paclitaxel and IC_50_ values calculated in HNF1A-overexpressing paclitaxel-resistant cells treated with different doses of triptolide. **g** Western blot showing the expression levels of the indicated proteins in HNF1A-overexpressing A549/PR cells treated with triptolide. Data are presented as the mean ± SD, *P < 0.05, **P < 0.01, ***P < 0.001. PR paclitaxel resistance, TPL triptolide.

Next, we aimed to ascertain whether triptolide could directly target HNF1A, an important protein involved in the development of paclitaxel resistance in NSCLC. We performed molecular docking analysis to assess the affinity of triptolide for HNF1A. The 3D structure and potential docking pockets of the HNF1A protein were shown in Supplementary Fig. S7a, b. The binding poses and interactions of triptolide with the HNF1A protein were demonstrated in Fig. 7a, revealing a highly stable interaction with a low binding energy of −7.048 kcal/mol. To confirm the direct modulatory effect of triptolide on the HNF1A protein, we analyzed their interaction by isothermal titration calorimetry (ITC). ITC experiments confirmed the direct high-affinity binding of triptolide to the HNF1A protein with a *K*_d_ value of 0.18 μM (Fig. 7b). Further Western blotting confirmed that triptolide significantly suppressed the expression of HNF1A protein in paclitaxel-resistant cells (Fig. 7c). Intriguingly, we noticed that the function of HNF1A as a transcription factor was significantly inhibited by triptolide, which was due to the reduced HNF1A protein expression in the nucleus (Fig. 7d). Similar changes in HNF1A protein levels were observed in tumor sections from A549 and A549/PR xenografts (Fig. 7e). In addition, HNF1A overexpression significantly impaired the triptolide-induced reduction in IC_50_ values in A549/PR and H460/PR cells, which supports the involvement of HNF1A in the triptolide-induced enhancement of paclitaxel sensitivity (Fig. 7f and Supplementary Fig. S7c). Moreover, forced HNF1A expression clearly attenuated triptolide-induced downregulation of SHH, PTCH1, GLI2, GLI3, and ABCB1 protein in paclitaxel-resistant cells, which solidly supported the crucial role of HNF1A in triptolide-mediated regulation of the Hedgehog signaling pathway (Fig. 7g and Supplementary Fig. S7d). Collectively, these results indicated that targeted inhibition of HNF1A expression was a vital factor in the ability of triptolide to reverse ABCB1-mediated paclitaxel resistance in NSCLC cells.

### HNF1A expression is positively correlated with SHH expression in clinical samples

**Fig. 8:**
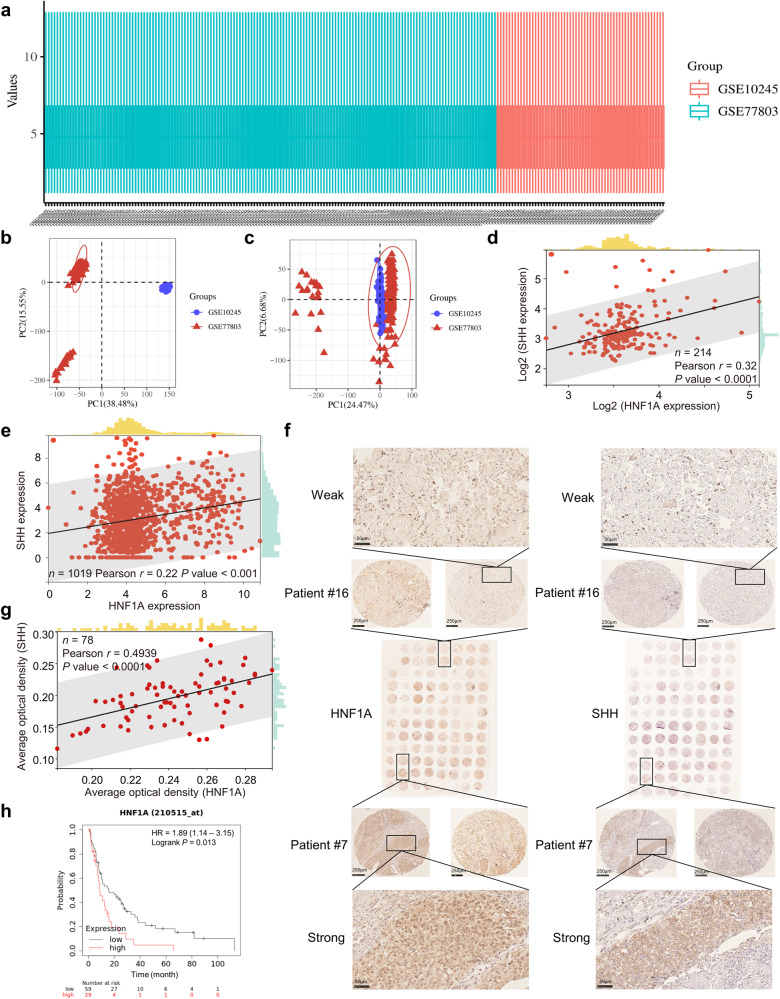
SHH expression in clinical tissue samples is positively correlated with HNF1A. **a** Different colors represented box plots of the data normalized for different GEO NSCLC data sets. **b** The PCA results before batch removal showed two separate data sets without any intersection. **c** The PCA results after batch removal showed that some data intersected together for subsequent analysis. **d** Results of two gene correlation analyses in the intersecting data. **e** Results of correlation analysis of two genes in the TCGA NSCLC dataset. **f** IHC staining for HNF1A and SHH was performed on two independent tumor microarrays (TMA1 and TMA2). Images of representative cases with strong or weak expression of HNF1A and SHH in TMA1 were enlarged in the figure. **g** Correlation analysis of the average optical density values of IHC staining for two genes in TMA1 and TMA2. Damaged samples were not considered for scoring. **h** Kaplan–Meier plot of post-progression survival in NSCLC patients receiving chemotherapy stratified by HNF1A expression status.

We next investigated whether the regulation of SHH by HNF1A was prevalent in NSCLC. The raw data of two gene expression profiles (GSE10245 and GSE77803) were downloaded from the GEO database of NCBI, and the data were normalized to produce box plots (Fig. 8a). PCA was performed on the dataset, as shown in Fig. 8b, c, to obtain the intersection. We then performed Pearson correlation analysis on the expression of HNF1A and SHH in 214 NSCLC samples, and the results showed a significant positive correlation (Fig. 8d). Similarly, the expression of HNF1A and SHH in NSCLC patients from the TCGA database also showed such an association (Fig. 8e).

Furthermore, 90 patients with NSCLC who underwent surgical treatment in our department were randomly enrolled in this study. The two most representative parts of the tumor tissues were selected to produce tissue microarrays (TMAs). As shown in Fig. 8f, IHC was applied to detect HNF1A and SHH protein levels in the TMAs, and representative images showed that HNF1A was mainly localized in the nucleus, while SHH protein was largely distributed in the cytoplasm. Importantly, Pearson correlation analysis revealed a significant positive correlation between the expression levels of HNF1A and SHH in the 78 nondamaged tissues (Fig. 8g). We then evaluated the correlation of HNF1A and SHH expression with clinicopathological characteristics. Interestingly, the results showed that high expression of HNF1A and SHH was significantly associated with advanced clinical stage and positive lymphatic invasion. The expression of these two genes was independent of the sex and age characteristics of the patients (Table 1). It supported that SHH expression is modulated by HNF1A and suggested an oncogenic function of HNF1A and SHH in NSCLC progression.

As previously noted, the Kaplan–Meier curves in Figs. 4a and 5d indicated that elevated expression levels of both HNF1A and SHH were associated with a poorer prognosis. Further online database (https://kmplot.com/analysis/) survival analysis showed that patients with high HNF1A expression among NSCLC patients receiving chemotherapy had poorer post-progression survival, suggesting that HNF1A expression was a reliable marker of chemotherapy efficacy in NSCLC patients (Fig. 8h). These results indicated that the HNF1A/SHH axis was an important potential target for triptolide or other drugs to improve chemosensitivity.

## Discussion

As a disruptor of microtubule kinetics, paclitaxel is commonly used for the treatment of various malignancies; however, most patients develop resistance with prolonged treatment. Paclitaxel resistance occurs frequently in patients with NSCLC on long-term chemotherapy and ultimately leads to a poor prognosis [11, 12]. Hence, there is an urgent need to explore the molecular mechanisms of paclitaxel resistance to discover new targets and drugs to address this problem. In the present study, we identified triptolide, a TCM monomer, capable of reversing ABCB1-mediated paclitaxel resistance and elaborated on the mechanism: triptolide regulated the Hedgehog pathway and ABCB1 via the HNF1A/SHH axis.

Increased expression of ABC superfamily multidrug efflux transport proteins such as ABCB1/P-gp, ABCC/MRP1 and ABCG2/BCRP are the main drivers of paclitaxel resistance. Many in vivo and in vitro experiments have revealed that ABCB1 overexpression in particular types of cancer cells significantly decreases the intracellular concentration of paclitaxel by increasing its efflux, which ultimately decreases or abolishes the pharmacological efficacy [39, 40]. In this study, we found that human NSCLC cells chronically exposed to paclitaxel can acquire paclitaxel resistance and exhibited a significant elevation in the expression of ABCB1. This suggested that our therapeutic approach based on suppression of ABCB1 mechanisms would increase the sensitivity to paclitaxel in resistant patients.

Accordingly, the search for an effective and nontoxic agent that downregulates ABCB1 expression to protect against NSCLC paclitaxel resistance is an important goal. Notably, traditional Chinese medicines (TCMs) are highly effective and safe, and several recent studies have shown their ability to reverse multidrug resistance [19, 20, 41]. TCM agents that can be used to reverse resistance can be classified as TCM monomers, synthetic monomers, analogs or derivatives with the ability to target multiple cellular pathways. Here, we investigated triptolide, a TCM monomer possessing a variety of biological functions that is extracted from *Tripterygium wilfordii* Hook F [42]. Reportedly, triptolide can inhibit NSCLC cell proliferation and metastasis [43]. We defined the function of triptolide in enhancing the sensitivity of NSCLC to paclitaxel through in vitro and in vivo experiments and found that it was achieved by blocking the expression of ABCB1. The ABCB1 gene was affected by triptolide at the transcriptional level in a dose-dependent manner. In addition, some studies have documented the ability of triptolide to increase sensitivity to different chemotherapeutic drugs in some human cancers through other mechanisms. For example, one of our earlier studies showed that triptolide can reverse paclitaxel resistance in lung adenocarcinoma through the NF-κB pathway [44]. Deng et al. reported that triptolide increased the sensitivity of breast cancer cells to doxorubicin by reducing the expression of ATM and suppressing the response to DNA damage [24]. Another study revealed that triptolide significantly sensitized cells to cytotoxicity induced by cisplatin, etoposide and epirubicin by inhibiting the Nrf2 signaling pathway [25]. Therefore, it is essential to investigate the role of triptolide in attenuating chemoresistance and its mechanisms in human pan-cancer in depth.

Previously, multiple studies have shown that some critical signaling pathways in cells can be involved in the development of multidrug resistance in cancer cells through the regulation of ABCB1. For example, the Hippo signaling pathway could influence regorafenib resistance in hepatocellular carcinoma (HCC) by modulating ABCB1 [45]. The NF-κB transcription factor inhibits apoptosis and induces drug resistance in cancer cells. Inhibition of NF-κB significantly reduced ABCB1 mRNA and protein expression in HCT15 cells [46]. Astragali radix, a well-known TCM, can trigger ABCB1 expression through activation of the Nrf2-mediated signaling pathway [47]. Intriguingly, some articles have reported that the Hedgehog pathway can directly regulate ABCB1 expression. Chao et al. found that activation of the Hedgehog pathway mediated chemoresistance by increasing drug efflux in an ABC transporter (e.g., ABCB1)-dependent manner [32]. HhAntag691, a Hedgehog pathway inhibitor, significantly suppressed the expression of ABCB1 [48]. Coincidentally, our RNA-seq analysis revealed the profiles of DEGs followed by KEGG enrichment analysis and found that the Hedgehog signaling pathway was involved in the reversal of the paclitaxel resistance process mediated by triptolide. As expected, it was experimentally confirmed that triptolide could affect ABCB1 expression by blocking the Hedgehog pathway. This finding also suggested that triptolide is a potent Hedgehog inhibitor.

In addition, current research on the reversal of drug resistance via the Hedgehog pathway mainly focuses on targeting the GLI family. Aberrant expression of the Hedgehog signaling pathway transcription factor Gli1 has been found to be involved in the modulation of ABCB1 in ovarian cancer [49]. However, our RNA-seq analysis revealed that it was not GLI1 but rather GLI2 and GLI3 that were activated in paclitaxel-resistant NSCLC cells, potentially due to alterations in the tumor microenvironment. Notably, PTCH1 is a key receptor of the Hedgehog pathway and a multidrug transporter protein involved in chemotherapeutic drug efflux. Unlike the ABC transporter family, PTCH1 uses proton motive force to efflux drugs [50]. In our results, PTCH1 was activated in paclitaxel-resistant cells, and its expression was significantly reduced after triptolide treatment. This is one of the mechanisms by which triptolide blocks the efflux of paclitaxel. Given this, we focused on the SHH gene, whose post-secretion binding to PTCH1 is the initial step in the activation of the Hedgehog signaling pathway [51]. ELISA results confirmed that the amount of SHH secreted in paclitaxel-resistant cells was influenced by triptolide treatment. Further rescue experiments showed that sustained activation of SHH is critical for maintaining paclitaxel resistance in NSCLC.

Identifying markers of chemoresistance is crucial for individualized cancer treatment, especially for NSCLC patients, who present variable responses to chemotherapy agents, limiting survival [52]. Hepatocyte nuclear factor 1-alpha (HNF1A) is a transcription factor with a wide range of functions. More specifically, this protein binds to promoter sequences required for hepatocyte-specific gene transcription and regulates functions related to cholesterol, bile acids and lipoprotein metabolism [53]. In recent years, there have been many reports on HNF1A-mediated regulation of drug resistance in cancer therapy. For example, Chen et al. found that HNF1A transcriptionally activated the expression of p53 binding protein 1 (53BP1) and that inhibition of HNF1A enhanced oxaliplatin resistance [54]. Eguchi et al. reported that HNF1A expression could reflect resistance to anti-colorectal cancer drug therapy, and inhibition of its expression could boost anti-colorectal cancer drug sensitivity [55]. In our study, HNF1A was identified as a novel transcription factor of SHH that regulates the expression of Hedgehog pathway components and potentiates ABCB1-mediated paclitaxel resistance. The HNF1A/SHH axis is a crucial junction involved in triptolide-induced reversal of paclitaxel resistance. Notably, according to the study from Lu et al., HNF1A overexpression could enhance sensitivity to gemcitabine by inhibiting ABCB1 transcription in pancreatic cancer [56], suggesting that the role of HNF1A in drug resistance is context dependent. Thus far, triptolide has been shown to directly target proteins such as XPB [57] and ADAM10 [58]. It was reported that knockdown of ADAM10 significantly reduced the IC_50_ values of adriamycin and paclitaxel in MDA-MB-231 cells [59]. Given the pivotal role of HNF1A in conferring chemoresistance, this study employed HNF1A as a target for triptolide molecular docking simulations. It elucidates the molecular mechanism underlying triptolide-mediated regulation of HNF1A expression, providing valuable insights into the therapeutic potential of this traditional Chinese medicine monomer.

Furthermore, the creation of the artificial intelligence (AI)-powered AlphaFold has shocked our community, and its latest version is supported by an innovative machine learning approach that integrates physical and biological knowledge about protein structure [60]. In a work published in Nature in 2020, scientists developed the first computational method capable of predicting protein structure with near experimental accuracy [61]. AlphaFold’s structure prediction capabilities are also believed to hold the promise of transforming the process of discovering novel and effective drugs [62, 63]. We employed AlphaFold2 to predict the protein structure of HNF1A and subsequently conducted molecular docking analysis with triptolide. However, molecular docking done based on AlphaFold predictions is inherently rigid. We further performed ITC experiments to confirm that triptolide can target binding to HNF1A protein.

In conclusion, this study reveals a novel mechanism underlying the ability of triptolide to reverse paclitaxel resistance: it inhibits HNF1A/SHH axis, thereby blocking the Hedgehog signaling pathway and ABCB1 expression and reducing paclitaxel efflux. This study paves the way for the therapeutic application of triptolide and provides hope for individualized treatment and prognosis improvement for chemotherapy-resistant NSCLC patients.

### Supplementary information

Supplementary Tables

Supplementary Figure S1

Supplementary Figure S2

Supplementary Figure S3

Supplementary Figure S4

Supplementary Figure S5

Supplementary Figure S6

Supplementary Figure S7

Supplementary figure legend
