# Supplementary material for: Targeted inhibition of the HNF1A/SHH axis by triptolide overcomes paclitaxel resistance in non-small cell lung cancer
Source: Acta Pharmacol Sin. 2024 Jan 16;45(5):1060–76. doi: 10.1038/s41401-023-01219-y (PMC11053095; doi:10.1038/s41401-023-01219-y)
Supplement: Supplementary file 1 — Supplementary Tables [file 41401_2023_1219_MOESM1_ESM.docx]

**Supplementary Table 1.** The sequence of siRNAs used in this study.

| **siRNA** | **Sequence (5'-3')** | **Purpose** |
| --- | --- | --- |
| siHNF1A#1 | Forward: GGUCCUACGUUCACCAACATT | Knockdown |
|  | Reverse: UGUUGGUGAACGUAGGACCTT |  |
| siHNF1A#2 | Forward: CGAAGAUGGUCAAGUCCUATT | Knockdown |
|  | Reverse: UAGGACUUGACCAUCUUCGTT |  |
| siHNF1B#1 | Forward: CAGUCAGCACCUUGACGAATT | Knockdown |
|  | Reverse: UUCGUCAAGGUGCUGACUGTT |  |
| siHNF1B#2 | Forward: CCUUAGUGGAGGAAUGCAATT | Knockdown |
|  | Reverse: UUGCAUUCCUCCACUAAGGTT |  |
| siMYB#1 | Forward: CCUGUAGCGUUACAUGUAATT | Knockdown |
|  | Reverse: UUACAUGUAACGCUACAGGTT |  |
| siMYB#2 | Forward: GACGAACUGAUAAUGCUAUTT | Knockdown |
|  | Reverse: AUAGCAUUAUCAGUUCGUCTT |  |
| siHOXD9#1 | Forward: CCGCCGAGUUCGCCUCGUGUA | Knockdown |
|  | Reverse: UACACGAGGCGAACUCGGCGG |  |
| siHOXD9#2 | Forward: GCACCCUCAGCAACUACUATT | Knockdown |
|  | Reverse: UAGUAGUUGCUGAGGGUGCTT |  |
| siHOXD10#1 | Forward: GAACAGAUCUUGUCGAAUATT | Knockdown |
|  | Reverse: UAUUCGACAAGAUCUGUUCTT |  |
| siHOXD10#2 | Forward: GCUGCAUGUAUUCUGAUAATT | Knockdown |
|  | Reverse: UUAUCAGAAUACAUGCAGCTT |  |
| siSHH#1 | Forward: GGUGUAAGGACAAGUUGAA | Knockdown |
|  | Reverse: UUCAACUUGUCCUUACACC |  |
| siSHH#2 | Forward: CGAGAUGUCUGCUGCUAGU | Knockdown |
|  | Reverse: ACUAGCAGCAGACAUCUCG |  |
| siABCB1#1 | Forward: CUAAUAGAAGUGAUAUCAA | Knockdown |
|  | Reverse: UUGAUAUCACUUCUAUUAG |  |
| siABCB1#2 | Forward: CGCUACUGAAGCAAUAGAA | Knockdown |
|  | Reverse: UUCUAUUGCUUCAGUAGCG |  |

| **Antibodies** | **Identifier** | **Purpose** |
| --- | --- | --- |
| SHH | Abcepta Cat# AP21229a | WB |
| SHH | Proteintech Cat# 20697-1-AP | IHC |
| GLI2 | Proteintech Cat# 18989-1-AP | WB |
| GLI3 | Abcam Cat# ab181130 | WB |
| PTCH1 | Abcepta Cat# AP71781 | WB |
| HNF1A | Abcam Cat# ab272693 | WB IHC |
| ABCB1 | Abcam Cat# ab170904 | WB IHC IF |
| ABCC1 | Abcam Cat# ab233383 | WB |
| ABCG2 | Abcam Cat# ab207732 | WB |
| LRP | Abcam Cat# ab175239 | WB |
| Myc-Tag | Cell Signaling Technology Cat# 2276S | WB |
| Anti-FLAG | Cell Signaling Technology Cat# 8146S | WB |
| β-Actin | ABclonal Cat# AC038 | WB |

**Supplementary Table 2.** The information of antibodies used in this study.

**Supplementary Table 3.** The sequence of primers used in this study.

| **Primer** | **Sequence (5'-3')** | **Purpose** |
| --- | --- | --- |
| SHH | Forward: CCGAGCGATTTAAGGAACTCACC | qPCR |
|  | Reverse: AGCGTTCAACTTGTCCTTACACC |  |
| ABCB1 | Forward: GCTGTCAAGGAAGCCAATGCCT | qPCR |
|  | Reverse: TGCAATGGCGATCCTCTGCTTC |  |
| GLI2 | Forward: GTCAGAGCCATCAAGACCGAGA | qPCR |
|  | Reverse: GCATCTCCACGCCACTGTCATT |  |
| GLI3 | Forward: TCAGCAAGTGGCTCCTATGGTC | qPCR |
|  | Reverse: GCTCTGTTGTCGGCTTAGGATC |  |
| PTCH1 | Forward: GCTGCACTACTTCAGAGACTGG | qPCR |
|  | Reverse: CACCAGGAGTTTGTAGGCAAGG |  |
| HNF1A | Forward: AGACGCTAGTGGAGGAGTGCAA | qPCR |
|  | Reverse: GGCAAACCAGTTGTAGACACGC |  |
| HNF1B | Forward: CCCAGCAAATCTTGTACCAGGC | qPCR |
|  | Reverse: ACCTCAGTGACCAAGTTGGAGC |  |
| HOXD9 | Forward: AGGAGGAGAAGCAGCATTCGCA | qPCR |
|  | Reverse: CTTTCTCCAGCTCAAGCGTCTG |  |
| HOXD10 | Forward: TGGCTGAGGTCTCCGTGTCCA | qPCR |
|  | Reverse: GCACCTCTTCTTTCTGCCACTC |  |
| MYB | Forward: GGGAACAGATGGGCAGAAATCG | qPCR |
|  | Reverse: GCTGGCTTTTGAAGACTCCTGC |  |
| SHH-promoter-primer | Forward: CCCGCCCACCTTTATCTTAGG | ChIP-qPCR |
|  | Reverse: GAGAGAGGCTGCCTTTAGCA |  |

**Supplementary Table 4.** Liver index and function in mice treated with triptolide, paclitaxel, or both.

| **Group** | **Liver index**  **(mg/g)** | **ALT (U/L)** | **AST (U/L)** | **ALP (U/L)** |
| --- | --- | --- | --- | --- |
| A549 (control) | 35.7±1.7 | 75±11 | 130.9±23.3 | 111.5±7.4 |
| A549 (PTX) | 36.8±2.6 | 95.6±18.7^*^ | 282.2±12.9^***^ | 120±10.6 |
| A549/PR (PTX) | 35.2±3.2 | 100.7±19^*^ | 242.95±31.8^***^ | 117.2±6 |
| A549/PR (TPL) | 34.7±3.6 | 70.7±17 | 285.9±29.1^***^ | 101±10.6 |
| A549/PR (PTX+TPL) | 37.7±3.1 | 139.6±31.2^***^ | 395.5±47.2^***^ | 123.2±11.5 |

PTX, paclitaxel; TPL, triptolide; ALT, alanine aminotransferase; AST, aspartate aminotransferase; ALP, alkaline phosphatase;

Results are expressed as the mean ± SD of six mice in each group.

**P* < 0.05, ****P* < 0.001 compared to control group (Dunnett’s *t*-test).

**Supplementary Table 5.** Kidney index and function in mice treated with triptolide, paclitaxel, or both.

| **Group** | **Kidney index**  **(mg/g)** | **Urea (mmol/L)** | **Cr (μmol/L)** | **UA (μmol/L)** |
| --- | --- | --- | --- | --- |
| A549 (control) | 6.3±0.2 | 8.3±0.5 | 86.3±11.4 | 112.2±8.8 |
| A549 (PTX) | 6.7±0.5 | 8.1±0.6 | 74±15.8 | 113.7±16.2 |
| A549/PR (PTX) | 6.7±0.4 | 7.7±0.7 | 89.5±14.6 | 108.6±17.6 |
| A549/PR (TPL) | 6.6±0.4 | 8.2±0.5 | 73.3±14.5 | 115.7±14.2 |
| A549/PR (PTX+TPL) | 6.8±0.4 | 8.3±0.3 | 81.3±15 | 102.7±14.2 |

PTX, paclitaxel; TPL, triptolide; Cr, creatinine; UA, uric acid;

Results are expressed as the mean ± SD of six mice in each group.

*P* > 0.05 compared to control group (Dunnett’s *t*-test).
