# Supplementary figures and images for: Targeted inhibition of the HNF1A/SHH axis by triptolide overcomes paclitaxel resistance in non-small cell lung cancer
Source: Acta Pharmacol Sin. 2024 Jan 16;45(5):1060–76. doi: 10.1038/s41401-023-01219-y (PMC11053095; doi:10.1038/s41401-023-01219-y)

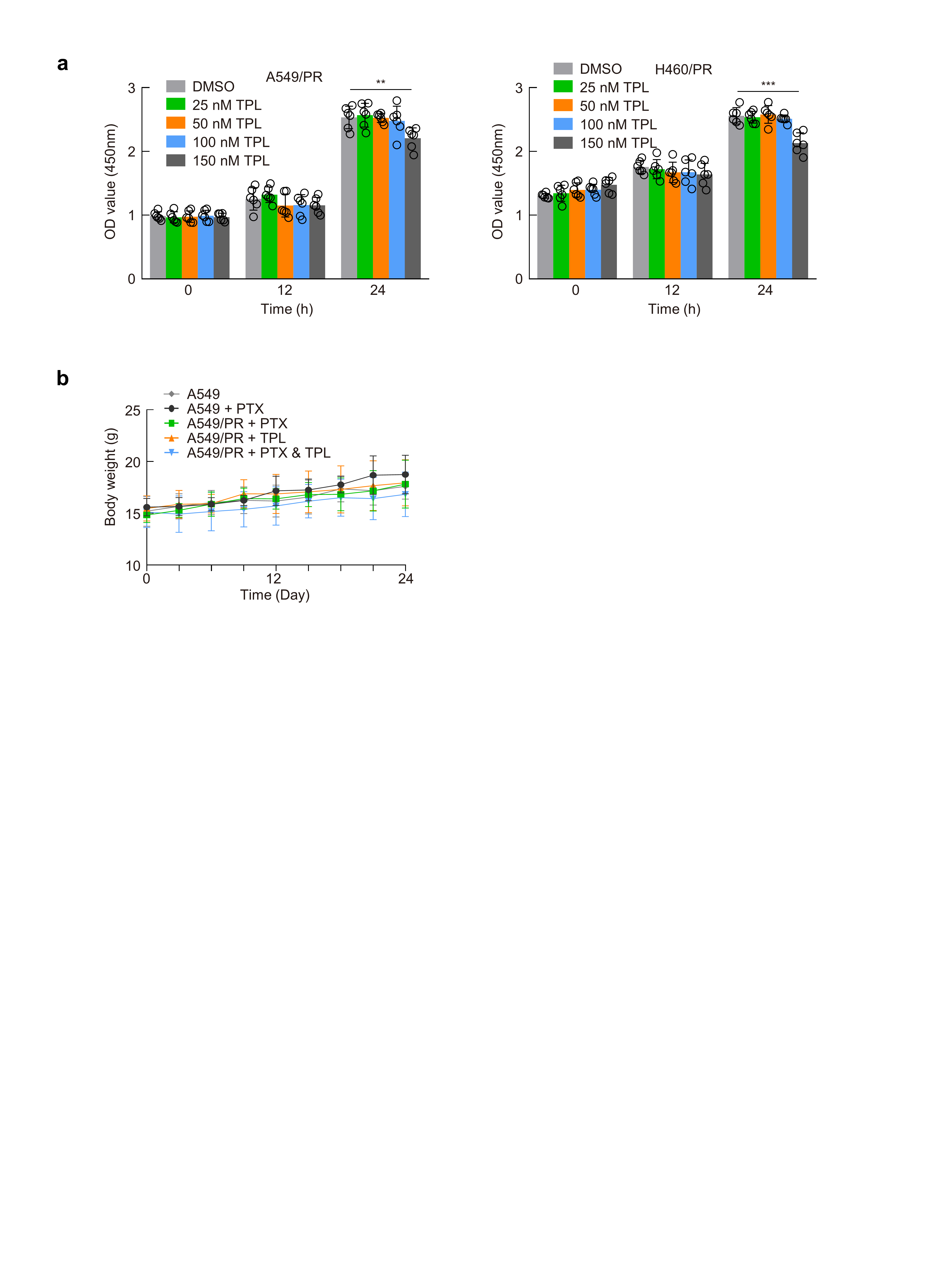

Supplement: Supplementary file 2 — Supplementary Figure S1 [file 41401_2023_1219_MOESM2_ESM.tif]

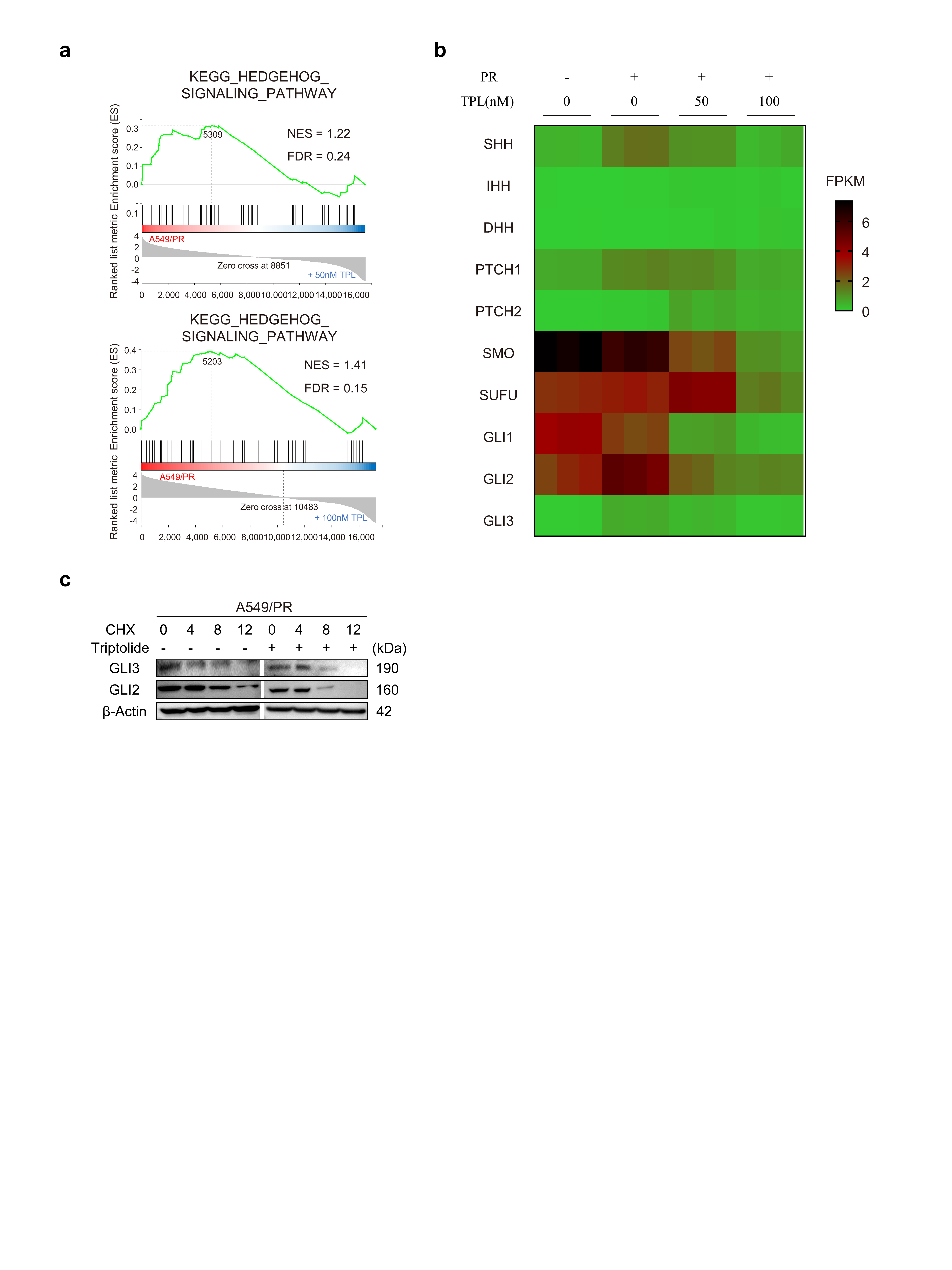

Supplement: Supplementary file 3 — Supplementary Figure S2 [file 41401_2023_1219_MOESM3_ESM.tif]

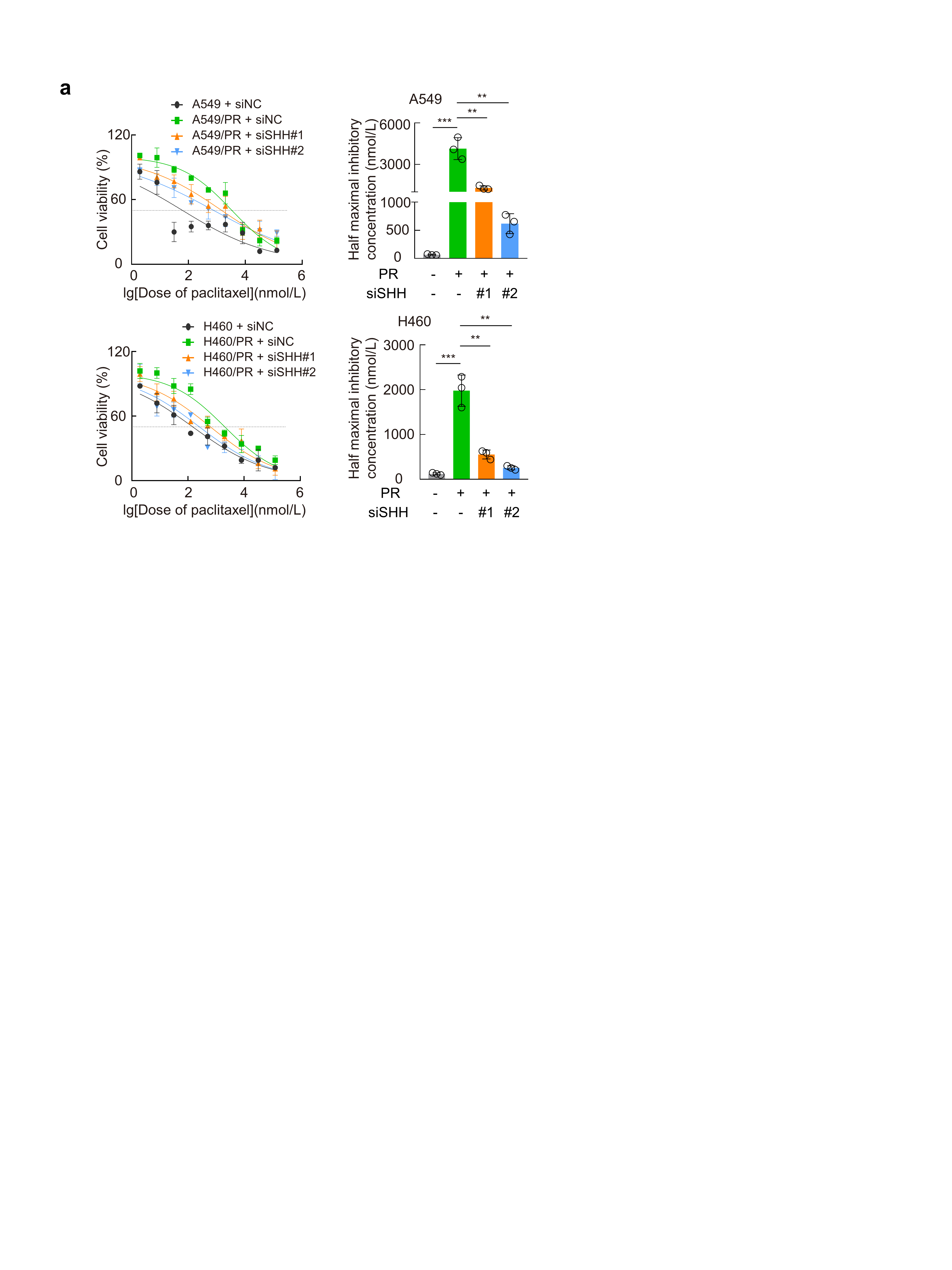

Supplement: Supplementary file 4 — Supplementary Figure S3 [file 41401_2023_1219_MOESM4_ESM.tif]

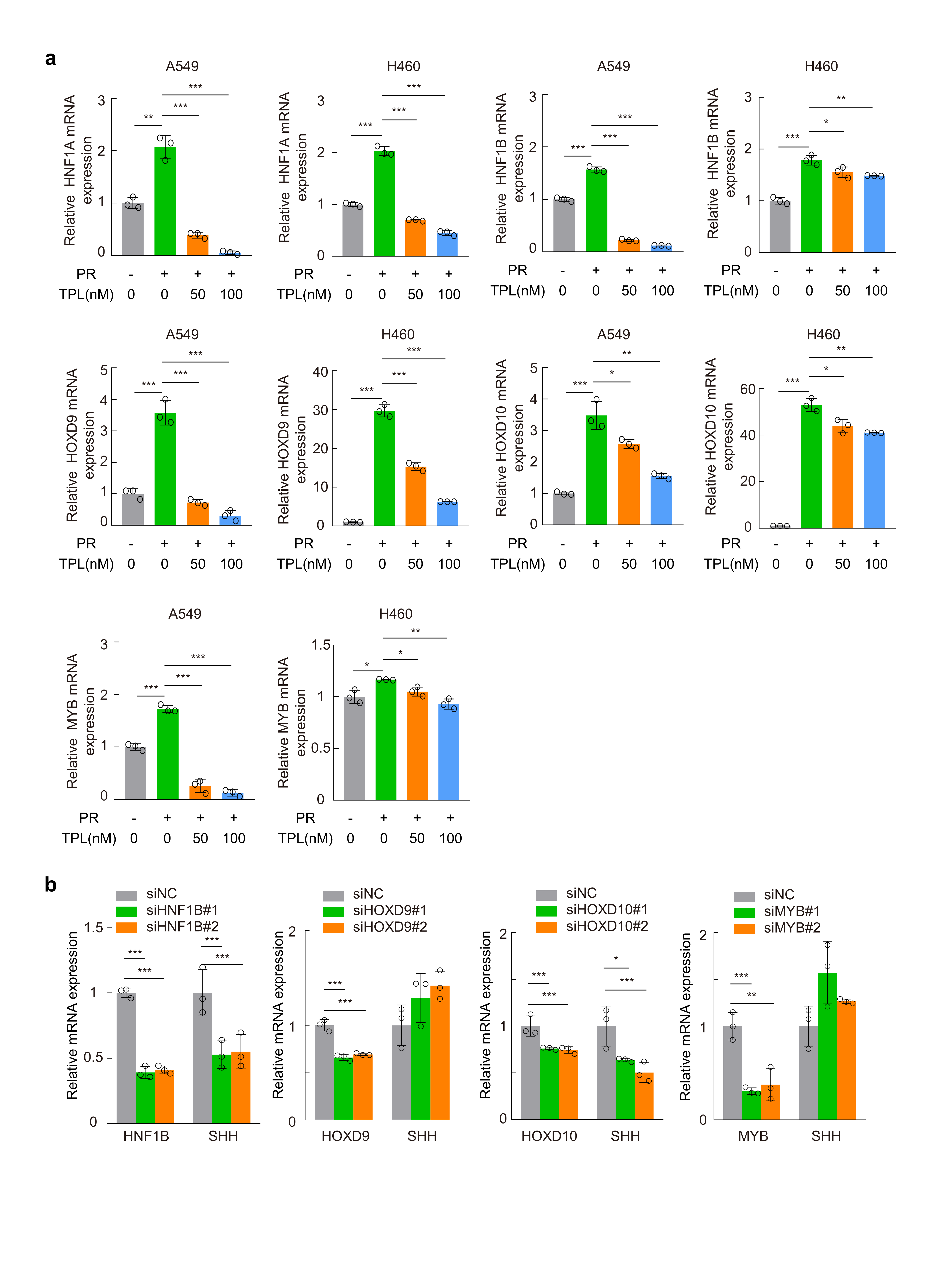

Supplement: Supplementary file 5 — Supplementary Figure S4 [file 41401_2023_1219_MOESM5_ESM.tif]

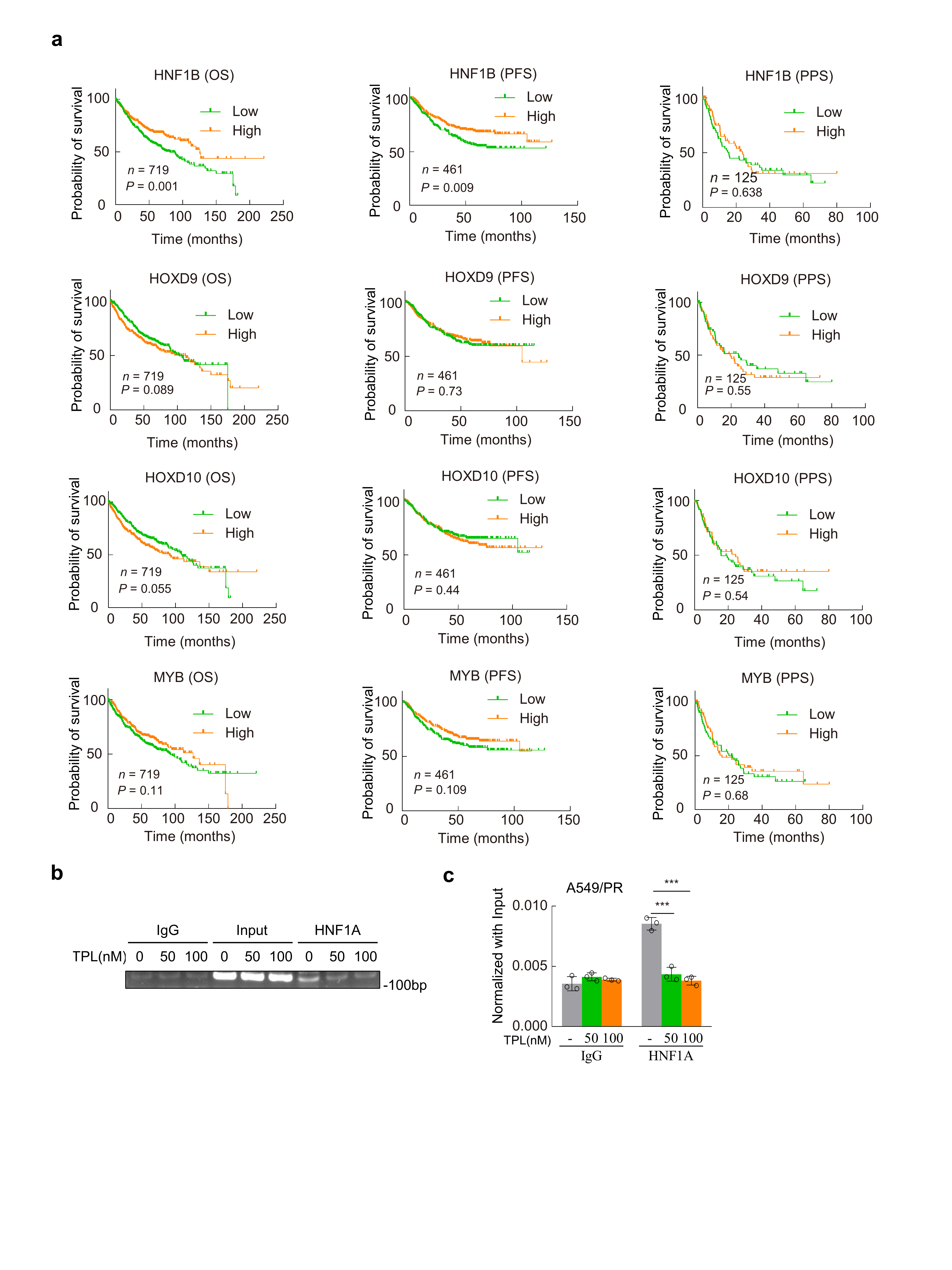

Supplement: Supplementary file 6 — Supplementary Figure S5 [file 41401_2023_1219_MOESM6_ESM.tif]

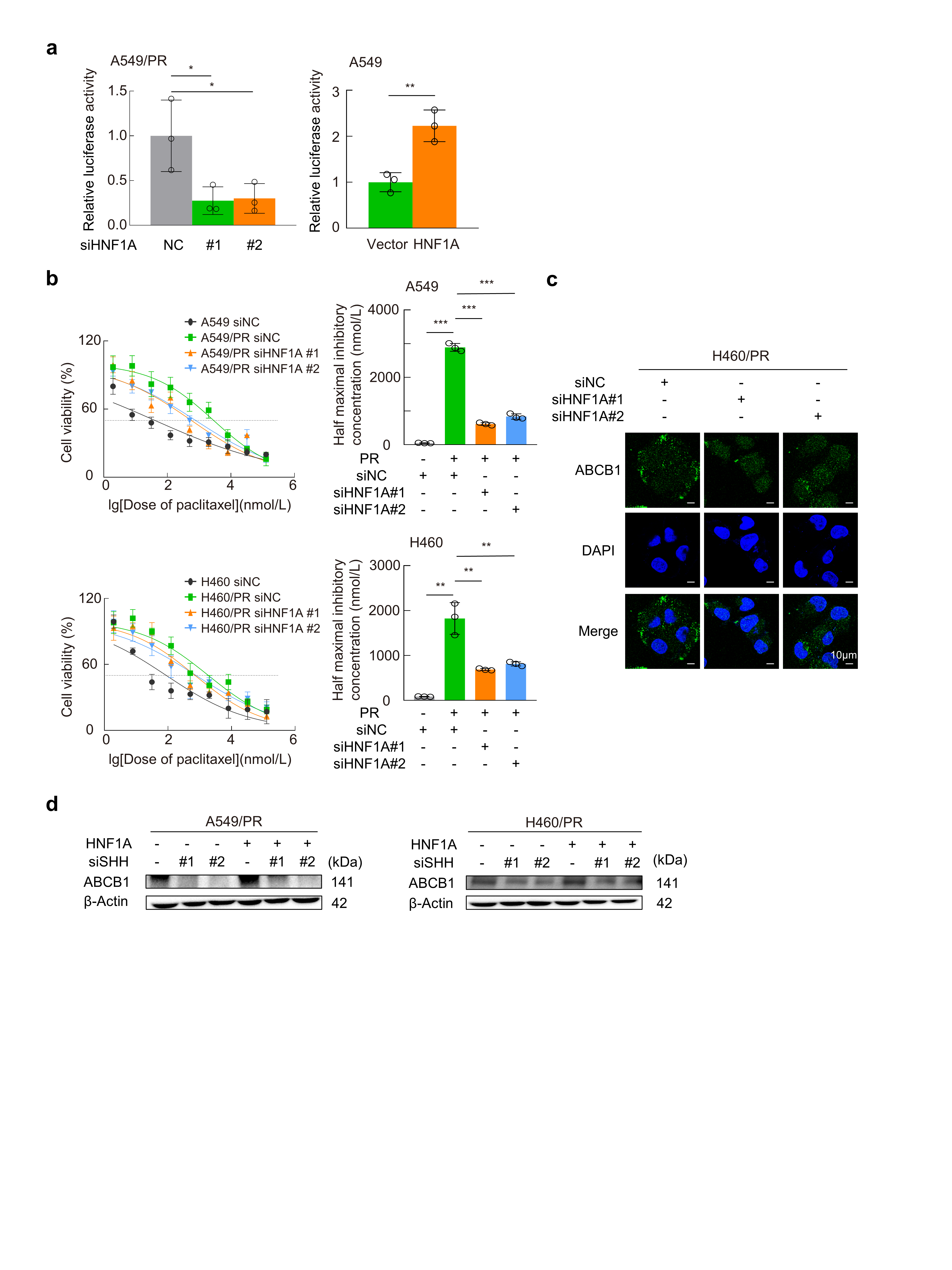

Supplement: Supplementary file 7 — Supplementary Figure S6 [file 41401_2023_1219_MOESM7_ESM.tif]

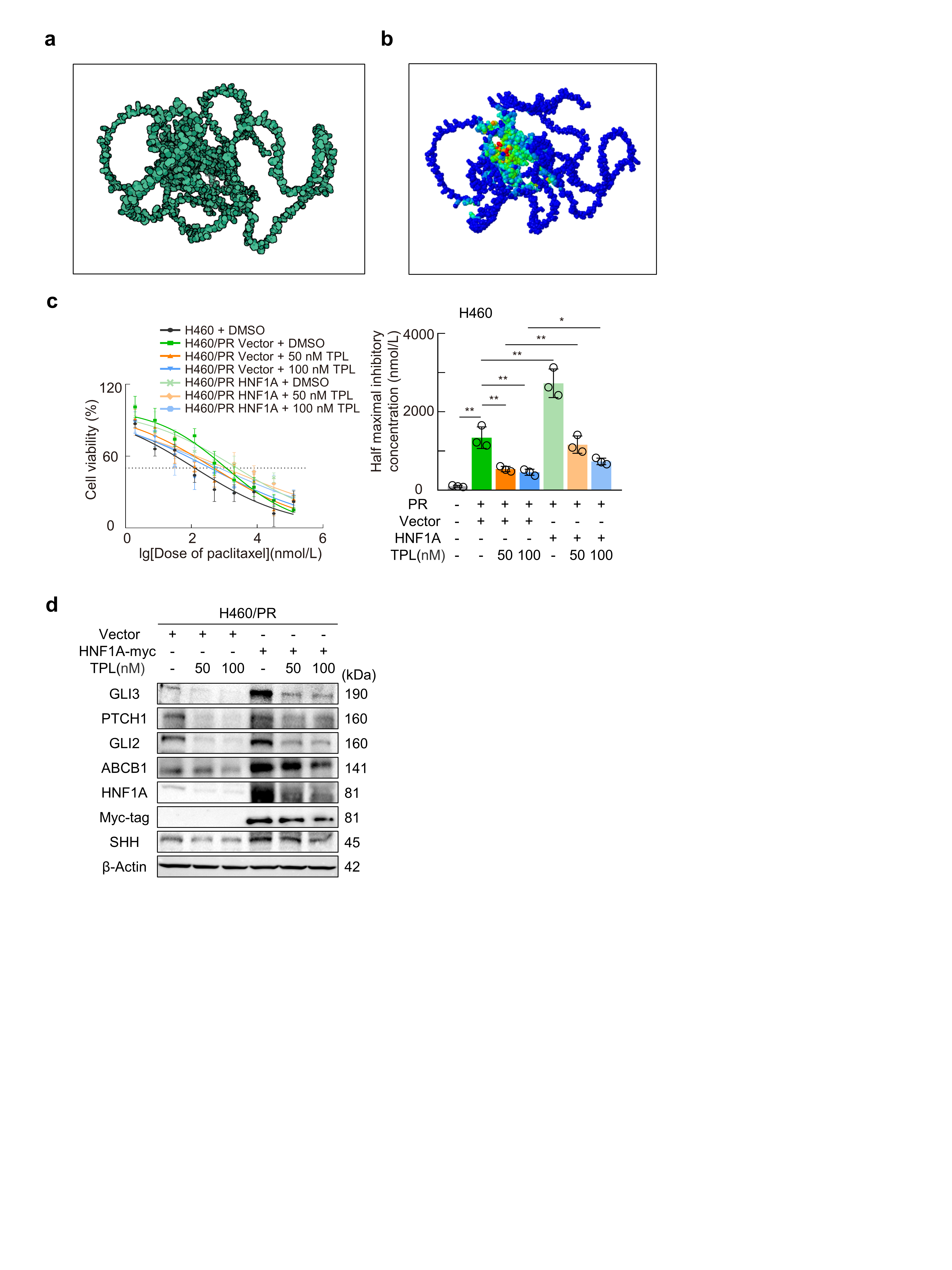

Supplement: Supplementary file 8 — Supplementary Figure S7 [file 41401_2023_1219_MOESM8_ESM.tif]
