# Supplementary material for: Targeted inhibition of the HNF1A/SHH axis by triptolide overcomes paclitaxel resistance in non-small cell lung cancer
Source: Acta Pharmacol Sin. 2024 Jan 16;45(5):1060–76. doi: 10.1038/s41401-023-01219-y (PMC11053095; doi:10.1038/s41401-023-01219-y)
Supplement: Supplementary file 9 — Supplementary figure legend [file 41401_2023_1219_MOESM9_ESM.docx]

Figure S1. Triptolide reverses ABCB1-mediated paclitaxel resistance, related to Figure 2. (a) The CCK-8 assay revealed dose-dependent proliferative alterations in A549/PR and H460/PR cells following 24 h exposure to varying concentrations of triptolide. (b) The weight of mice was measured every 3 days during drug administration. Data are presented as the mean ± SD, PR, paclitaxel resistance. TPL, triptolide.

Figure S2. The sonic Hedgehog pathway is involved in the triptolide-mediated reversal of paclitaxel resistance, related to Figure 3. (a) GSEA was used to evaluate the difference in the Hedgehog signaling pathway between the A549/PR and A549/PR groups treated with 50 or 100 nM triptolide. (b) Heatmaps show the expression differences of the indicated genes among A549, A549/PR, and A549/PR cells treated with 50 or 100 nM triptolide. (c) Western blot assay showed that the expression levels of GLI2 and GLI3 proteins in A549/PR cells were affected by 50 nM triptolide after different time treatments of cycloheximide at 50 µg/mL. TPL, triptolide. CHX, cycloheximide.

Figure S3. SHH expression is a key factor in induced paclitaxel resistance, related to Figure 4. (a) Changes in the IC_50_ of paclitaxel were observed after knocking down SHH in paclitaxel-resistant cells. Data are presented as the mean ± SD, ** *P <* 0.01, *** *P <* 0.001. PR, paclitaxel resistance.

Figure S4. RT‒qPCR of four other candidate TFs, related to Fig. 5. (a) RT-qPCR detection of mRNA expression levels of five candidate transcription factors HNF1A, HNF1B, HOXD9, HOXD10, and MYB before and after triptolide treatment. (b) Changes in SHH mRNA expression after knockdown of four candidate transcription factors in A549/PR cells. Data are presented as the mean ± SD, * *P <* 0.05, ** *P <* 0.01, *** *P <* 0.001. PR, paclitaxel resistance. TPL, triptolide.

Figure S5. Kaplan‒Meier analysis of four other candidate TFs, related to Fig. 5. (a) Kaplan‒Meier curve reveals the OS, PFS, and PPS of LUAD patients stratified by HNF1B, HOXD9, HOXD10, and MYB expression in the KM-Plotter data set. (b) ChIP assays were conducted on A549/PR cells treated with triptolide, utilizing specific HNF1A or IgG antibodies. The results were semi-quantitatively analyzed through agarose gel electrophoresis. (c) The results were quantitatively analyzed using real-time qPCR. Data are presented as the mean ± SD, *** *P <* 0.001.

Figure S6. Downregulation of HNF1A inhibits ABCB1 expression, reversing paclitaxel resistance (related to Fig. 6). (a) Dual luciferase reporter gene assay for relative expression levels of 8× GliBS luciferase in A549/PR cells knocking down HNF1A and in A549 cells overexpressing HNF1A. (b) Cell viability was detected by CCK-8 assays, and the dose‒response curves for paclitaxel in HNF1A knockdown cells were fitted. These histograms show IC_50_ values based on the curves. (c) Immunofluorescence was used to assess the location and abundance of ABCB1 protein in H460/PR cells transfected with siNC, siHNF1A#1, and siHNF1A#2. (d) Repression of SHH blocks the upregulation of ABCB1 protein expression induced by HNF1A overexpression in paclitaxel-resistant cells, as evidenced by Western blot analysis. Data are presented as the mean ± SD, * *P <* 0.05, ** *P* < 0.01, *** *P* < 0.001. PR, paclitaxel resistance.

Figure S7. Triptolide specifically inhibits HNF1A, as demonstrated in Fig. 7. (a) The three-dimensional structure of the HNF1A protein. (b) The binding pockets of the HNF1A protein. (c) Dose-response curves for paclitaxel were determined using CCK-8 cell viability assays, and IC_50_ values were calculated in HNF1A-overexpressing H460/PR cells treated with triptolide. (d) Western blot shows protein expression levels in HNF1A-overexpressing H460/PR cells treated with triptolide.
